# Machine learning toward advanced energy storage devices and systems

**DOI:** 10.1016/j.isci.2020.101936

**Published:** 2020-12-13

**Authors:** Tianhan Gao, Wei Lu

**Affiliations:** 1 Department of Mechanical Engineering, University of Michigan, Ann Arbor, MI 48109, USA; 2 Department of Materials Science & Engineering, University of Michigan, Ann Arbor, MI 48109, USA

**Keywords:** Applied Computing, Energy Storage, Materials Design

## Abstract

Technology advancement demands energy storage devices (ESD) and systems (ESS) with better performance, longer life, higher reliability, and smarter management strategy. Designing such systems involve a trade-off among a large set of parameters, whereas advanced control strategies need to rely on the instantaneous status of many indicators. Machine learning can dramatically accelerate calculations, capture complex mechanisms to improve the prediction accuracy, and make optimized decisions based on comprehensive status information. The computational efficiency makes it applicable for real-time management. This paper reviews recent progresses in this emerging area, especially new concepts, approaches, and applications of machine learning technologies for commonly used energy storage devices (including batteries, capacitors/supercapacitors, fuel cells, other ESDs) and systems (including battery ESS, hybrid ESS, grid and microgrid-containing energy storage units, pumped-storage system, thermal ESS). The perspective on future directions is also discussed.

## Graphical Abstract

**Figure:**
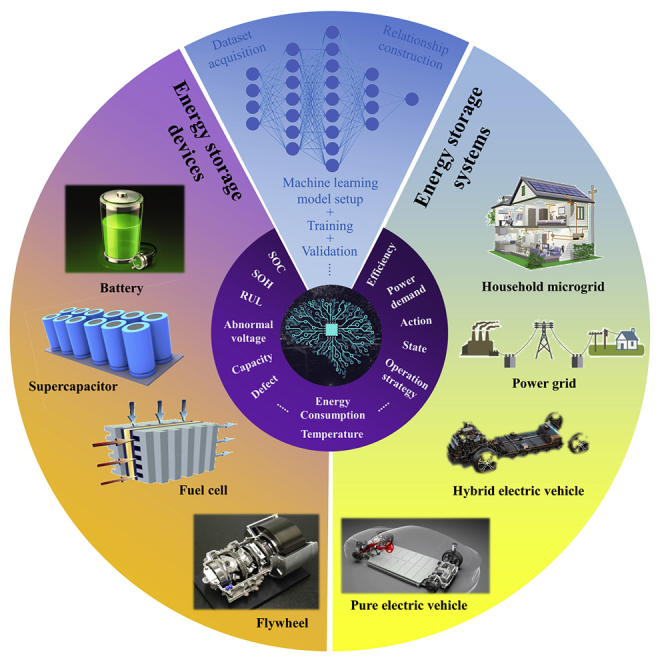

## Introduction and overviews

With economic growth, global energy consumption increases significantly during the last decade. For instance, the global electricity and fossil fuel consumption increased by 19.44% and 9.14%, respectively from 2010 to 2017, according to the statistics data from the International Energy Agency. These have aggravated the issue of energy shortage and CO_2_ emission. The amount of CO_2_ emission has increased by 22.2% from 2010 to 2019. Improving the efficiency of energy usage and promoting renewable energy become crucial. The increasing use of consumer electronics and electrified mobility drive the demand for mobile power sources, which stimulate the development and management of energy storage devices (ESDs) and energy storage systems (ESSs). The increasing complexity of ESDs and ESSs and the large amount of front-end data pose significant challenges to traditional models and algorithms. New state-of-the-art technology is needed to address the issues faced by the traditional approaches for higher accuracy, more efficiency, and better optimization.

Machine learning (ML), coupled with big data, has been flourishing in recent years. Integrating human knowledge into machine learning (Deng et al., 2020) has achieved functions and performance not available before and facilitated the interaction between human beings and machine learning systems, making machine learning decisions understandable to humans. Beyond the field of computer and data sciences such as computer vision, natural language processing, image recognition, and search engine, machine learning is increasingly used in the field of physics (Carleo et al., 2019; Dunjko and Briegel, 2018), chemistry (Goh et al., 2017; Panteleev et al., 2018), biology (Silva et al., 2019; Zitnik et al., 2019), engineering (Flah et al., 2020; Kim et al., 2018; McCoy and Auret, 2019), and materials science (Morgan and Jacobs, 2020). Besides the above-mentioned disciplines, machine learning technologies have great potentials for addressing the development and management of energy storage devices and systems by significantly improving the prediction accuracy and computational efficiency. Several recent reviews have highlighted the trend. The work in (Zhang et al., 2019a) reviewed the use of deep learning technologies on prognostics and health management (PHM), which include fault detection, diagnosis, and prognosis in application domains including batteries. The work in (Hu et al., 2020; Meng and Li, 2019) reviewed the application of different approaches, including physics-based (model-based) approaches, data-driven approaches, and hybrid approaches, on the PHM and lifetime prognosis for batteries. The work in (Ng et al., 2020) reviewed the application of machine learning on the estimation of state of charge and state of health for batteries. The work in (Erick and Folly, 2020) reviewed the application of reinforcement learning for the management of grid-tied microgrid energy systems, specifically aiming at control problems. The work in (Chen et al., 2020; Gu et al., 2019) reviewed the application of machine learning in the field of energy storage and renewable energy materials for rechargeable batteries, photovoltaics, catalysis, superconductors, and solar cells, specifically focusing on how machine learning can assist the design, development, and discovery of novel materials. These reviews mainly focus on the application of certain types of machine learning algorithms in a specific subarea. Recognizing that the field of energy storage device and system as well as machine learning is broad, a more comprehensive review is needed to provide a better representation and guidance of the relevant state-of-the-art research and development. A unique aspect of this review is to provide a coverage of machine learning in both device and system level applications.

In this paper, we provide a comprehensive review of recent advances and applications of machine learning in ESDs and ESSs. These include state estimation, lifetime prediction, fault and defect diagnosis, property and behavior analysis, modeling, design and optimization for ESDs, as well as modeling and optimization of the control strategy for ESSs. The structure of this paper is organized as follows: first, we discuss the status and challenge that the current ESDs and ESSs are facing. Then, we introduce the major machine learning technologies that have been used in the field of energy storage. Next, we present how to apply machine learning for ESDs. After that, we introduce the application of machine learning for ESSs. Finally, we provide a summary and perspective on future directions.

### Development and challenges of current energy storage devices and systems

ESDs can store energy in various forms (Pollet et al., 2014). Examples include electrochemical ESD (such as batteries, flow batteries, capacitors/supercapacitors, and fuel cells), physical ESDs (such as superconducting magnets energy storage, compressed air, pumped storage, and flywheel), and thermal ESDs (such as sensible heat storage and latent heat storage based on phase change materials). Batteries alone have various types, such as lithium-ion, sodium-ion, lead-acid, nickel–metal hydride, and zinc-air batteries. Representative flow batteries are vanadium flow, zinc-based flow, and polysulfide-halide flow batteries. Commonly used fuel cells include alkaline, polymer membrane exchange, solid oxide, and microbial fuel cells (Mejia and Kajikawa, 2020). Batteries are widely used in automobiles, consumer electronics, mobile power, aeronautics and astronautics, medical equipment, and security systems. Supercapacitors are used in applications that require high current and frequent charge/discharge cycles, such as automobiles, energy harvesting, and consumer electronics. Fuel cells are attractive for medium to heavy duty transport and are used in automobiles, electricity generation, aeronautics, and astronautics. The flywheel is mainly used for uninterruptible power supply in the power grid to provide backup power instantaneously, as well as in some types of vehicles. Both flywheel and compressed air can serve for storing redundant electricity and supply the electricity during the peak of demand. The pumped storage is mainly used for generating electricity. Thermal ESDs are mainly used for heat storage and reuse in buildings and industrial processes and storage of the solar energy for electricity generation. The typical ESD parameters include specific energy, specific power, storage capacity, response time, efficiency, charge-discharge rate, lifetime, capital/operational cost, heat sensitivity, maintenance, etc (Pollet et al., 2014).

The goal for current ESD development can be grouped into five categories: (1) lowering the cost, (2) improving the performance and efficiency, (3) ensuring the usage safety, (4) promoting the reliability and durability, and (5) reducing the environmental impact. Achieving these goals rely on accurate ESD modeling that guides the performance analysis and design. One associated challenge is the identification of model parameters. Appropriate ESD design, including choice of structural parameters, material selection, as well as designing operational strategies, is critical in ensuring the target cost, performance, efficiency, durability, and safety. A challenge is to systematically optimize the design for various conditions. Besides, monitoring and predicting the ESD status such as the state of charge, state of health, and capacity of a battery is vital for the management system to perform and adjust its controlling strategy to maximize the performance, prolong the lifetime, and ensure the safety of an ESD. The status is often difficult to directly measure and predict. A challenge is to distill and acquire useful information from a large measured dataset.

Appropriate design and optimization of ESS is critical to achieve high efficiency in energy storage and harvest. An ESS is typically in the form of a grid or a microgrid containing energy storage units (a single or multiple ESDs), monitoring units, and scheduling management units. Representative systems include electric ESS and thermal ESS. Specifically, the electric ESS mainly includes battery ESS, battery/supercapacitor/fuel cell hybrid ESS, hydraulic ESS, flying wheel ESS, and compress air ESS. ESSs are widely used in transportation (especially pure electrical vehicles and hybrid electrical vehicles), consumer electronics, grids and microgrids, buildings, aeronautics and astronautics applications, etc. The goal of ESS development is to achieve high energy storage capacity, high power distribution ability, high operation and energy usage efficiency, long durability, and low system cost. A main challenge for current ESSs is the selection and adjustment of control strategy based on the status of each unit and the energy demand.

### Overview of machine learning technologies

#### Representative types of machine learning algorithms

##### Unsupervised learning

Unsupervised learning performs learning on unlabeled dataset and is typically used in the problems of clustering. A commonly used algorithm is the *k*-mean clustering algorithm (such as the *k*-nearest neighbor algorithm (*k*-NN)). This algorithm first randomly sets *k* initial centroids for *k* clusters with the samples assigned to the closest cluster centroids and then moves the centroids to minimize the cost function (e.g., the distance between the samples and the centroids). The learning process is terminated when the cost function is minimized or the maximum iteration is reached. Hierarchical clustering is another widely used algorithm, which calculates the similarity between two data points (quantified by the Euclidean distance), between two datasets (quantified by a distance calculated by the single linkage, complete linkage, or average linkage method), or between a data point and a dataset, and clusters the two units having the highest similarity. The learning process is terminated when the number of clusters decreases to a given value. Density-based spatial clustering of applications with noise (DBSCAN), proposed in the work of (Ester et al., 1996), is also a commonly used clustering algorithm. This algorithm groups the data points that are closely packed (described by the scanning radius, *ε*, and the minimum number of points required to form a dense region, *MinPts*) and marks the data points located in the low-density regions as outliers. The learning process is terminated when all the data points are scanned. Other clustering algorithms include the Gaussian mixture model and the mean shift algorithm.

##### Supervised learning

Supervised learning performs learning on labeled data, which is widely used in the problems of classification and regression. Linear regression (LR), polynomial regression, and exponential regression are the fundamental regression algorithms to construct a direct relationship between the independent and dependent variables. Besides, the Gaussian processing regression (GPR) is a type of regression algorithm that is gaining increasing attention. The GPR assumes that the sample obeys a Gaussian random process distribution (*GP*) rather than a parametric form, which is determined by its mean and covariance function (as indicated in (Equations 1, 2, and 3), where ***x*** and ***x****'* denote two different input vectors, *m*(***x***) is the mean function, and *κ*(***x***, ***x****'*) is the covariance function). Specifically, the covariance function (kernel function) has multiple formats such as linear kernel, squared exponential kernel, Matérn kernel, periodic kernel, or a compound format of multiple types of kernel functions. During training with a labeled sample dataset, the hyperparameters in the function Equation (1) are optimized. The GPR has a higher estimation accuracy when dealing with non-linear relationships than the LR algorithm.

(Equation 1) $$f\left(x\right)=GP\left(m\left(x\right),\kappa \left(x,x^{\prime }\right)\right),$$

(Equation 2) m(x)=E(f(x)),

(Equation 3) $$\kappa \left(x,x^{\prime }\right)=E\left[\left(f\left(x\right)-m\left(x\right)\right){\left(f\left(x\right)-m\left(x\right)\right)}^{T}\right].$$

Support vector machine (SVM) is a representative kernel-based supervised learning algorithm. For solving classification problems on the linearly separable dataset (such as (***x***, ***y***), where ***x*** denotes the input sample vector and ***y*** denotes the labeled target vector), SVM uses two parallel hyperplanes (margin) to clearly separate the data. The decision boundary in the middle of the two parallel hyperplanes has a form shown in Equation (4), where ***w*** and ***b*** denote the weight and the bias parameter vector, respectively. The distance between the decision boundary and each hyperplane is 1/‖w‖for a normalized or standard dataset. For solving classification problems on the linearly inseparable dataset, SVM introduces a hinge loss function to the hyperplanes to reduce the classification errors, and the problem changes to minimizing the function *L* in Equation (5), where *n* denotes the sample number and *λ* denotes a regularization parameter. Specifically, SVM can also apply the kernel method (the decision boundary has a form shown in Equations (6) and (7), where *φ* is a mapping function) by using a kernel function such as those in Table 1 to transform the input vectors to a higher dimensional feature space (Hilbert space) and then uses hyperplanes to separate the data. SVM can also be used to solve regression problems (called SVR, or support vector regression). The interior point method (IPM), sequential minimal optimization (SMO), and stochastic gradient descent (SGD) are often employed for solving the parameters in the hyperplanes. Other commonly used SVM forms include support vector clustering (SVC) and Bayesian SVM. The relative vector machine (RVM) is also a kernel-based model and is based on the sparse Bayesian learning theory (Tipping, 2001, 2003). RVM is similar to SVM and has been used in the field of energy storage.

(Equation 4) $$w^{T}x+b=0,$$

(Equation 5) $$L=\left[\frac{1}{n}\sum_{i=1}^{n}\max\left(0,1-y_{i}\left(w^{\text{T}}x_{i}+b\right)\right)\right]+\lambda {\left\|w\right\|}^{2}.$$

(Equation 6) $$w^{T}\varphi \left(x\right)+b=0,$$

(Equation 7) $$\kappa \left(x_{i},x_{j}\right)=\varphi {\left(x_{i}\right)}^{T}\varphi \left(x_{j}\right).$$

**Table 1:** Commonly used kernel functions for vector machine algorithms

Kernel function name	Expression
Linear kernel	$\kappa \left(x_{i},x_{j}\right)=\left(x_{i}^{T}x_{j}\right)$
Polynomial kernel	$\kappa \left(x_{i},x_{j}\right)={\left(x_{i}^{T}x_{j}\right)}^{n}$
Laplacian kernel	$\kappa \left(x_{i},x_{j}\right)=\exp\left(-\left\|x_{i}-x_{j}\right\|/\sigma \right)$
Radial basis kernel (Gaussian kernel)	$\kappa \left(x_{i},x_{j}\right)=\exp\left(-{\left\|x_{i}-x_{j}\right\|}^{2}/2\sigma^{2}\right)$
Sigmoid kernel	$\kappa \left(x_{i},x_{j}\right)=\tanh\left[a\left(x_{i}^{T}x_{j}\right)-b\right],\;a,b>0$

Note: ***x***_*i*_ and ***x***_*j*_ denote two sample vectors, *n*, *a*, *b*, *σ* denote the parameters in the kernel function.

Decision tree (DT) is another supervised learning algorithm. DT firstly chooses features to form a root node and calculates the information gain (rate) of the features. The feature with the largest information gain is selected as the node feature, whereas child nodes are built based on different values of the feature. Further child nodes are generated in the same way for each child node until the information gain is small or there are no features to choose from. The DT commonly uses the ID3, C4.5, or CART algorithm to calculate and optimize the information gain, which is based on information entropy. The random forest (RF) algorithm, which is a type of ensemble learning containing multiple DT models, can be used to increase the robustness of a tree-based algorithm. The RF algorithm surveys the result of each DT model and chooses the result with the most votes as its result.

##### Deep learning

**Figure 1:**
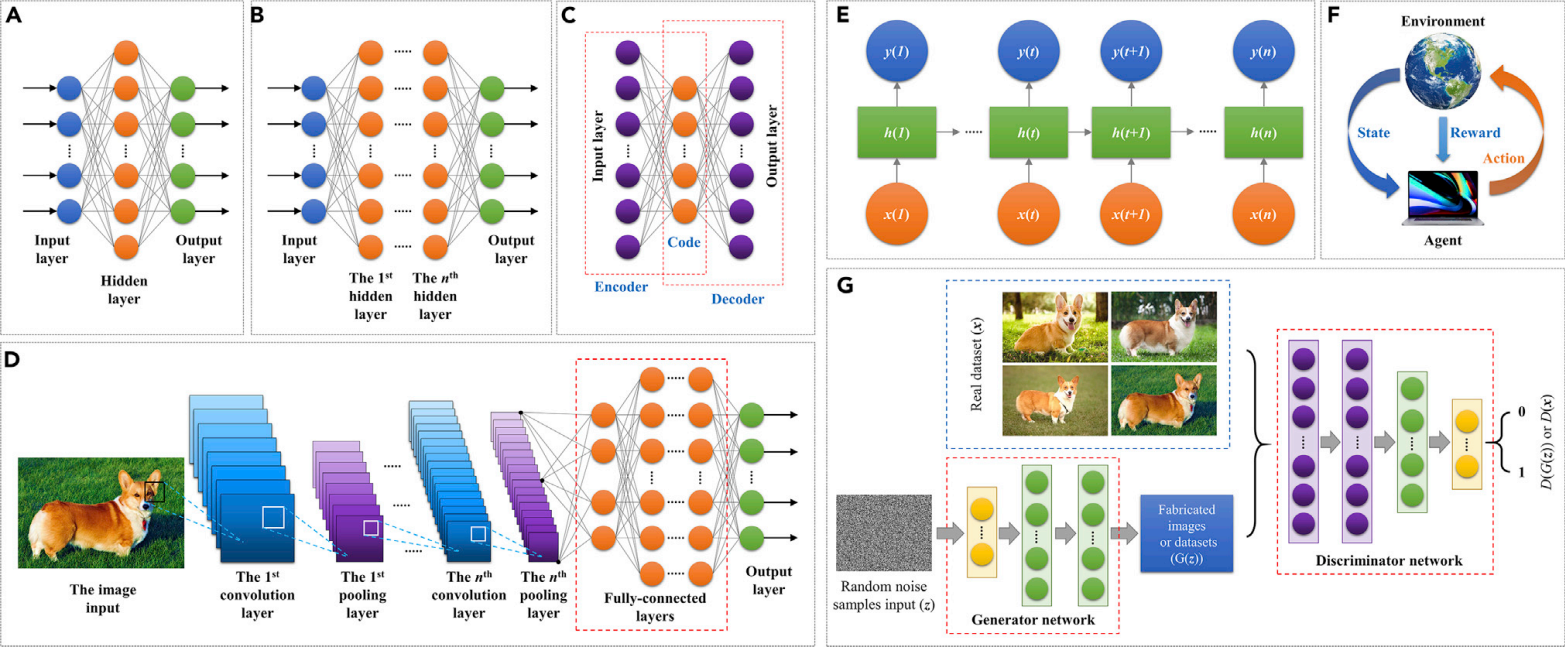
A schematic of commonly used machine learning models (A) Single-layer feed-forward neural network (SLFNN). (B) Deep neural network (DNN). (C) Auto encoder (AE). (D) Convolution neural network (CNN). (E) Recurrent neural network (RNN). (F) Reinforcement learning architecture. (G) Generative adversarial network (GAN).

Deep learning builds upon artificial neural networks (ANNs) and can be either unsupervised or supervised. It establishes a relation between the input and target parameters by non-linear functions and uses certain methods to calculate the function parameters. Deep learning receives increasing attention along with the development of big data mining and advanced computational technologies. A standard ANN model is the single-layer feedforward neural network (SLFNN), which contains an input layer, a hidden layer, and an output layer as shown in Figure 1A. An SLFNN can be described by Equation (8), where ***x*** denotes the input sample, *f*(***x***) denotes the SLFNN output, *f*_*a*_ denotes the activation function (e.g., Table 2), ***W*** is the weight, and ***b*** is the bias. A deep neural network (DNN) contains more than one hidden layer (Figure 1B). A DNN can be described by Equation (9). SLFNN and DNN are trained through backpropagation (an optimization algorithm based on gradient descendant to optimize the weight and bias in the neural network). The deep belief network (DBN) is another widely used form of ANN proposed in the work of (Hinton et al., 2006).

(Equation 8) $$y=f\left(x\right)=f_{a}\left(W_{0}^{\text{T}}f_{a}\left(W_{0}^{\text{T}}x+b_{0}\right)+b_{1}\right),$$

(Equation 9) $$f\left(x\right)=f_{a}\left(W_{i}^{\text{T}}f_{a}\left(W_{i-1}^{\text{T}}\ldots f_{a}\left(W_{0}^{\text{T}}x+b_{0}\right)\right)+b_{i}\right).$$

**Table 2:** Commonly used activation functions in deep learning

Name (*f*_*a*_)	Expression
Sigmoid function	$f_{a}\left(x\right)=1/\left(1+\exp\left(-x\right)\right)$
ReLu function	$f_{a}\left(x\right)=\max\left(0,x\right)$
Tanh function	$f_{a}\left(x\right)=\left(\exp\left(x\right)-\exp\left(-x\right)\right)/\left(\exp\left(x\right)+\exp\left(-x\right)\right)$

The extreme learning machine (ELM) proposed by (Huang et al., 2011) is a type of neural network with a single hidden layer. The weights and the biases are randomly defined, whereas the output layer contains no bias. During training, the weights of the output layer are transformed to a linear system for solving by a pseudo-inverse method. This is different from the typical ANN-based model, where the weight and bias parameters are obtained by the gradient descent algorithm.

Auto encoder (AE) is a neural network-based unsupervised learning algorithm, which contains an encoder and a decoder as shown in Figure 1C. The encoder maps the input dataset to the code (also known as latent variables or latent representation), whereas the decoder maps the code to the reconstructed original input. There are three types of commonly used AE variants, including the sparse AE, the denoising AE, and the contractive AE. Besides, multiple AE models combined with other algorithms (such as the clustering algorithms) can act as a deep learning model for machine learning.

The convolution neural network (CNN) is a deep learning model with a strong capability of performing feature distillation and representation learning. CNN is widely used for image recognition and object recognition. A typical CNN model contains convolutional layers, pooling layers, fully connected layers, and loss layers, as shown in Figure 1D. The convolutional layer uses convolutional kernels to sweep the input tensor or the tensor of previous convolutional layers to distill the feature information to form feature maps. The convolutional layer has a format shown in Equaitons (10) and (11) (Goodfellow et al., 2016), where $Z^{l+1}\left(i,j\right)$ denotes the (*i*^th^, *j*^th^) pixel in the output from the (*l*+1)^th^ convolutional layer (feature map), $Z_{k}^{l}$ denotes the input to the (*l*+1)^th^ convolutional layer (the *k*^th^ channel), *K* denotes the total number of channels in the *l*^th^ convolutional layer, *L*_*l+*1_ denotes the size of $Z^{l+1}$, $w_{k}^{l+1}\left(x,y\right)$ denotes the weight value of the (*x*^th^, *y*^th^) element in the convolutional kernel in the (*l*+1)^th^ convolutional layer, ***b*** denotes the bias vector (determined by the convolutional kernel), *f* denotes the size of the convolutional kernel, *s*_0_ denotes the stride number, and *p* denotes the padding number. The pooling layer selects features and filter the information, which usually has the form in Equation (12) (Estrach et al., 2014), where $P_{k}^{l+1}\left(i,j\right)$ is the output and *a* denotes the parameter that determines the pooling strategy (*a* = 1 denotes the average pooling; *a*→∞ denotes the max pooling). The max and average pooling strategies are often used. The convolutional layer and the pooling layer often appear alternatively to provide better data mining performance. The fully connected (FC) layer combines the distilled features non-linearly and send them to the output, which has a similar format as shown in Equations (8) and (9). Similar to SLFNN and DNN, CNN is typically trained by the backpropagation algorithm. To improve the training efficiency and model robustness, transfer learning (TL) and ensemble learning (EL) strategies are sometimes used for DNN and CNN models. TL trains a complex neural network to converge on small sets of training data. EL increases the accuracy and robustness of a neural network by combining multiple learning systems.

(Equation 10) $$Z^{l+1}\left(i,j\right)=\sum_{k=1}^{K}\sum_{x=1}^{f}\sum_{y=1}^{f}\left[Z_{k}^{l}\left(s_{0}i+x,s_{0}j+y\right)w_{k}^{l+1}\left(x,y\right)\right]+b,\;\left(i,j\right)\in \left\{0,1,\ldots L_{l+1}\right\},$$

(Equation 11) $$L_{l+1}=\left(L_{l}+2p-f\right)/s_{0}+1,$$

(Equation 12) $$P_{k}^{l+1}\left(i,j\right)={\left[\sum_{x=1}^{f}\sum_{y=1}^{f}Z_{k}^{l}{\left(s_{0}i+x,s_{0}j+y\right)}^{a}\right]}^{\frac{1}{a}}.$$

Recurrent neural network (RNN), as shown in Figure 1E, is widely used for processing time-series data. The input for each RNN block is a variable *x*(*t*) at each moment *t*. Each block has a hidden status *h*(*t*) and an output *y*(*t*). The *h*(*t*) is imported to the next unit and combines with *x*(*t*+1) to produce *h*(*t*+1) as well as the output *y*(*t*+1). This process allows an RNN to have a time-series memory. A trained RNN can predict the patterns of input time-series variables. Long short-term memory (LSTM), originally proposed by (Hochreiter, 1997), is a representative RNN architecture. Each LSTM block contains a forget gate (which can “forget” the “unimportant” inputs to the block, whereas strengthen the “important” inputs), which can solve the problem of gradient vanishing and exploding. This leads to better training performance for long time-series scenarios than the traditional RNN algorithm. Other RNN architectures include gate recurrent unit (GRU), which is a mutation type of LSTM (Cho et al., 2014), stacked RNN, bidirectional RNN, and reservoir computing (RC).

##### Reinforcement learning

Reinforcement learning (RL) is mostly used for an intelligent agent to choose actions that give the maximum cumulative reward during its interaction with the environment, building on the principle of Markov decision process. A typical RL model contains an environment and an agent. The agent learns actions in response to the environment based on the state of the environment, whereas the environment sends back reward to the agent, as shown in Figure 1F. RL can be divided into two categories—model-based and model-free RLs—depending on whether explicit modeling of the environment is required. Common RL algorithms include the following: (1) *Q*-learning that uses the quality values *Q*(*s*, *a*) stored in the *Q*-table to generate the action for the next step and then the quality value is updated based on Equation (13), where *α* denotes the learning rate, *γ* denotes the deduction factor, *R* denotes the reward, *a* and *s* denote the action and state in the current step, and *a'* and *s'* denote the action and state in the next step; (2) the deep *Q*-network (DQN) that applies deep learning algorithms (e.g., DNN, CNN, DT) to generate a continuous *Q* quality value in order to overcome the exponentially increasing computational cost of *Q*-learning; (3) the policy gradient algorithm that generates the next-step action based on the policy function (which is the quantification of state and action values at the current step) instead of the quality value such as *Q*; and (4) actor-critic algorithm that uses the actor to generate the next-step action based on the current-step state and then adjust its policy based on the score from the critic, whereas the critic uses the critic function to score the actor at the current step and then adjusts its scoring policy.

(Equation 13) $$Q\left(s,a\right)\leftarrow Q\left(s,a\right)+\alpha \left[R+\gamma \max_{a^{\prime }}Q\left(s^{\prime },a^{\prime }\right)-Q\left(s,a\right)\right],\;s\leftarrow s^{\prime }.$$

#### Data pre-processing

Data pre-processing is needed in many cases in order to achieve high training accuracy for ML models. This is because the amount of data for different types of datasets can vary significantly, causing an ML model to fail to capture the features of datasets with less data. Data compaction on the dataset is one of the widely used pre-processing methods. Principal component analysis (PCA) is often used to compact the data. The idea is to reduce the dimension of the dataset while keeping the main features within the dataset. PCA firstly calculates the correlation coefficient matrix (or the covariance matrix) of the pre-processing data matrix, then calculates the eigenvector of the correlation coefficient matrix (or the covariance matrix), and finally projects the data to the space formed by the feature vector. Besides PCA, AE is also widely used for data compaction. Other algorithms such as the undersampling algorithms based on clustering has also been used to compact the dataset.

In some application scenarios, the amount of data for certain types of dataset is limited, which can cause difficulties to train an ML model to achieve high learning accuracy (especially for the datasets containing complex features or ML models with complex structures such as a DNN with multiple hidden layers or a CNN with multiple convolutional and pooling layers). This is because the parameters of an ML model can hardly reach their optimized values during training (for ML models using optimization algorithms to acquire the model parameters) or be solved explicitly (for ML models that solve the model parameters analytically). It is important to have a large dataset with similar features but different details to train an ML model. To solve this problem, the generative adversarial network (GAN), an unsupervised learning proposed by (Goodfellow et al., 2014), is widely used. The GAN can generate or reconstruct dataset with features similar to the original inputs as well as to repair or enhance the quality of the dataset. A GAN typically contains a generator and a discriminator (two neural networks as shown in Figure 1G). The input to the generator is the noise vector (containing information of the real dataset), whereas the output from the generator is the fabricated dataset. The input to the discriminator is the fabricated dataset and the real dataset, whereas the output from the discriminator is the result showing whether the fabricated dataset is fake or real. The training principle for the GAN is to optimize the game function in Equation (14), which is quantified based on the theory of cross-entropy. Specifically, the generator firstly fabricates a dataset that can easily cause the discriminator to judge whether the dataset is generated or real. The generator keeps on training to reduce the difference between the fabricated and the real datasets based on the loss function *ℓ*_*G*_ in Equation (15), where ***z*** ∼ *p*(***z***) denotes the random noise samples obeying a random probability distribution *p*(***z***), *G*(***z***) denotes the generated datasets based on the random noise samples (the output datasets from the generator), and *D*(*G*(***z***)) denotes the judgment result of the fabricated datasets by the generator (the output scalar from the discriminator). At the same time, the discriminator keeps on training to distinguish the fabricated dataset and the real dataset based on the loss function *ℓ*_*D*_ in Equation (16), where ***x*** ∼ *p*_*data*_ denotes the real datasets obeying a certain distribution characteristics *p*_*data*_, and *D*(***x***) denotes the judgment result of the real datasets (the output scalar from the discriminator). The training of a GAN ends when the discriminator cannot distinguish whether the fabricated dataset by the generator is fake or real (i.e. the system optimization reaches the Nash equilibrium, D(x)=1/2). In addition to GAN, other types of algorithms such as the oversampling algorithm based on SMOTE (Chawla et al., 2002) are also widely used to regenerate datasets with similar features.

(Equation 14) $$\min_{G}\max_{D}E_{x∼p_{data}}\left[\log\;D\left(x\right)\right]+E_{z∼p\left(z\right)}\left[\log\left(1-D\left(G\left(z\right)\right)\right)\right],$$

(Equation 15) $$\ell_{G}=-E_{z∼p\left(z\right)}\left[\log\;D\left(G\left(z\right)\right)\right],$$

(Equation 16) $$\ell_{D}=-E_{x∼p_{data}}\left[\log\;D\left(x\right)\right]-E_{z∼p\left(z\right)}\left[\log\left(1-D\left(G\left(z\right)\right)\right)\right].$$

Besides data compaction and dataset construction, data normalization and data smoothing are also commonly used before training an ML model. Data normalization scales the values of the data to be within a certain range (typically within the range of (0,1)), which can accelerate the convergence of model parameters during training and increase the learning accuracy. The typical data normalization methods include the min-max normalization calculated by *x'*=(*x*-min(*x*))/(max(*x*)-min(*x*)) and the mean normalization calculated by *x'*=(*x*-mean(*x*))/(max(*x*)-min(*x*)). Data smoothing uses some algorithms to reduce the noise in the signal, which is important in reflecting the characteristics of the dataset and avoiding overfitting during training. The commonly used data smoothing algorithms include moving average, exponential mean average, Savitzky Laplacian smoothing, kernel smoother, Golay filter, and Kalman filtering.

## Approaches and applications of machine learning for ESDs

In this section, the application of machine learning for the development and management of energy storage devices is reviewed. We first introduce the three most commonly used types of ESDs, including batteries, capacitors/supercapacitors, and fuel cells. The problems that machine learning mainly focuses on are state estimation and prediction, lifetime prediction, property analysis and classification, fault discovery and diagnosis, as well as modeling, design, and optimization. The commonly used ML algorithms include unsupervised learning, supervised learning, and deep learning. For each application, the problem description, setup of the ML model, and resulting performance are discussed. Finally, the application of machine learning for the flow battery and flywheel are introduced.

### Application of machine learning for batteries

#### Battery state estimation

The battery state mainly includes state of charge (SOC), capacity, and state of health (SOH). Specifically, SOC is quantified by the ratio of the releasable capacity of a battery over its rated capacity. SOH is quantified by the ratio of the maximum releasable capacity over the rated capacity. SOC and SOH are important parameters for the battery management system (BMS) to choose appropriate controlling strategies to improve the performance and to ensure the safety and lifetime of the battery system (Murnane and Ghazel, 2017).

We first show the application of supervised learning for battery state estimation. For the application of regression methods, the work in (Richardson et al., 2017) applied GPR to forecast the SOH of batteries. The GPR model contains a compound kernel function composed of two types of Matérn kernel functions with *ν* of 5/2 and 3/2 (Ma5+Ma3). Specifically, they applied an integer number of cycles to be the input to the GPR model and applied the corresponding measured capacity to be the output from the GPR model. Their results showed that the compound kernel function has the advantage of capturing complex behaviors. Using multi-output GPs, the correlation between the data from different cells can be effectively explored. This can improve the forecasting performance, whereas the calculation efficiency may be reduced as a result of the need to handle a large amount of output. Besides, they also applied GPR to estimate the *in-situ* capacity of Li ion batteries (Richardson et al., 2018). The GPR model contains a Matérn (*ν* of 5/2) kernel function. The work in (Sahinoglu et al., 2017) applied GPR models (including regular GPR model, recurrent GPR model, and autoregressive recurrent GPR model) to estimate the SOC of Li ion batteries. The GPR models contain a squared exponential kernel function. They set the voltage, current, and temperature at the moment *k* to be the input to the regular GPR model; the voltage, current, and temperature at the moment *k* and the SOC at the moment (*k* −1) to be the input to the recurrent GPR mode; and the voltage, current, and temperature at the moment *k* and the voltage, current, temperature, and SOC at the moment (*k* −1) to be the input to the autoregressive recurrent GPR model. They set the SOC to be the output of the three GPR models and applied a dataset of 2000 data points to train their models. The results show that the recurrent GPR model has a better estimation accuracy than the regular GPR model.

Besides the above-mentioned regression approaches, a random forest regression model is applied to estimate on-line SOH (Li et al., 2018). Specifically, features are extracted (including the relative capacity values with *ΔV* intervals in a specific voltage region) from the voltage-capacity curves. The associated SOH values are used to construct the training dataset. The RF model contains 500 decision trees and provides high accuracy in SOH prediction with a mean squared error (MSE) below 1.3%. In another work (Andre et al., 2013), a dual filter consisting of a standard Kalman filter (SKF) (Kalman, 1960) and an unscented Kalman filter (UKF) (Julier and Uhlmann, 1997) is employed to estimate the SOC and SOH for Li-ion batteries. An SVR is used to couple with the dual filter. High accuracy in SOC estimation with an error below 1% is achieved.

**Figure 2:**
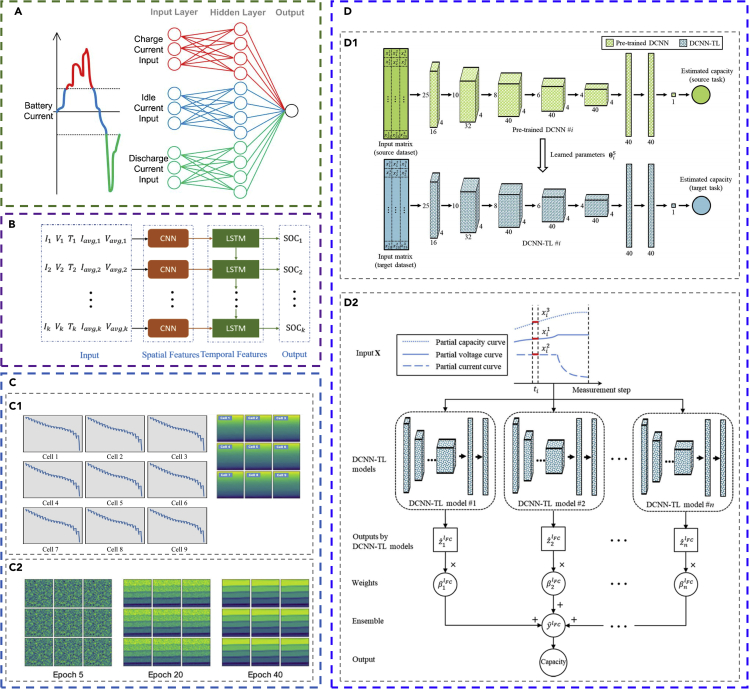
Examples of the application of machine learning for battery state estimation (A) The structure of the load-classification neural network model. Reprinted from (Tong et al., 2016). The input data are divided into three subsets based on the three types of battery behavior (charging, idling, and discharging). Each subset is imported into a specific split of the input layer (distinguished by three colors). (B) The structure of the CNN-LSTM hybrid model. Reprinted from (Song et al., 2019). The input parameters are firstly imported into the CNN module, which has a layer with six filters of length three. Then the CNN-processed data are imported to the LSTM module, which has a layer with hidden nodes. (C) The application of GAN-CLS for generating the required data rather than running experiments to train the bidirectional LSTM model. Reprinted from (Zhang et al., 2020). (C1) Converting the vector data (the voltage curves with pulse characteristics of nine battery cells) to 2D images for importing to the discriminator after feature mining by a CNN. (C2) The generated image representing the voltage data with pulse characteristics during the training of the GAN-CLS. Note that random data can be generated as voltage data after 40 epochs. (D) A schematic of the transfer learning and ensemble learning for DCNN models to estimate the battery capacity. Reprinted from (Shen et al., 2020). (D1) An illustration of the transfer learning process. (D2) An illustration of the ensemble learning model, which contains *n* DCNN-TL models.

ANN-based models are widely used for battery state estimation. DNN is used to estimate the SOC of Li-ion batteries (Chemali et al., 2018). The voltage, temperature, average current, and average voltage of the battery at moment *t* are used as the input of the DNN, whereas the SOC value at moment *t* is the output. The DNN shows high accuracy in predicting the SOC with an average mean absolute error (MAE) of 1.10% at 25°C and 2.17% at −20°C. In another work, a radial basis function neural network (RBFNN) model is applied to estimate the capacity and internal resistance of Li-ion batteries (Zhou et al., 2020b). The model contains one hidden layer, with the input being the three characteristic voltage points collected during the series discharging after normalization and with the output being the capacity and internal resistance. Among the data collected from 108 cells (acquired by simulation), 50% of them is used to train the RBFNN model. The capacity and internal resistance are predicted with an error below ±5% and ±0.4%, respectively. A load-classifying neural network (NN) model is also developed to estimate SOC (Tong et al., 2016). The NN classifies the input vectors (including the extracted features of current, voltage, and time) into three categories (charging, idling, and discharging, as shown in Figure 2A) based on the current and trains the three sub-NNs in parallel. Thus, the load-classifying NN has a simpler training procedure, a broader choice of training data, and smaller computational cost. The output of the load-classifying NN is the estimated SOC (after filtering). The model is trained with the load profile of a vehicle's driving cycle. It is shown that the average absolute estimation error of SOC is 3.8%. Extreme learning machine (ELM) is used for SOH estimation of LiNMC batteries on-line (Pan et al., 2018). The input for the ELM is the ohmic internal resistance and the polarized internal resistance (suggested by the result of health indicator extraction), whereas the output is capacity degradation. The ELM provides on-line SOH estimation with a maximum error below 2.5%.

CNN and RNN are used to estimate the battery state based on complex feature datasets with time-series characteristics. A combination of CNN and LSTM networks are employed to estimate the SOC of Li-ion batteries (Song et al., 2019). The CNN-LSTM structure is shown in Figure 2B, where the CNN is used for pattern recognition and spatial feature distillation, whereas the LSTM is used to process the time-series data (learn the temporal features of the battery dynamic evolution). A total of 24,815 groups of data points is acquired from experiments to train the CNN-LSTM system. The input in each group include the current, voltage, temperature, average current, and voltage, whereas the output is the time-dependent SOC estimation. The trained CNN-LSTM can estimate the SOC with the overall root-mean-square error (RMSE) and MAE below 2% and 1%, respectively. In a GRU-based RNN model to estimate the SOC (Xiao et al., 2019), the Nadam and AdaMax ensemble optimizers (Dozat, 2016; Kingma and Ba, 2014) are used to optimize the model parameters during the GRU-RNN training. The input for the RNN model includes the time-series of current, voltage, and temperature after normalization, whereas the output from the RNN model is the time series of estimated SOC. A dropout algorithm (with a dropout probability of 0.2) is used to prevent overfitting during training. Trained with 80% of the 18 simulated data subsets (a total sample number of 1,26,356), the GRU-RNN model gives high accuracy in SOC estimation with an average RMSE of 1.13% on the testing dataset. In another RNN model to estimate SOH (You et al., 2017), the input for the LSTM include the current and voltage at each present moment and the output parameter at each previous moment. The output parameter of the LSTM at each present moment is imported to the pooling layer and finally to the regression layer (which finally constructs the relationship between the current, voltage, and SOH at each present moment). A vanilla neural network is coupled to assist in alleviating the sensitivity to noise. Trained with 20 sets of experimental data, the model shows an average validation error on capacity estimation lower than 0.0765 Ah (RMSE of 2.46%). Studies have also used a combination of GAN-CLS and bidirectional LSTM (Bi_LSTM) to predict SOC (Zhang et al., 2020). The GAN-CLS is used for generating labeled datasets to train the Bi_LSTM model, which can reduce the amount of experimental running. The battery charging/discharging data (OCV curves) are converted into images (as shown in Figure 2C), and then the GAN-CLS model is employed to generate the corresponding data based on the images. After that, the newly generated data are used to train the Bi_LSTM model to predict the OCV characteristics. The results show that the modeling and training/testing time is significantly reduced. The MSE and average MSE of prediction are less than 0.0025 and 0.0013, respectively.

The strategy of transfer learning and ensemble learning is gaining increasing attention for improving the training efficiency and robustness of the ML model. Deep convolutional neural networks coupled with ensemble learning and transfer learning (DCNN-ETL) are used to estimate the capacity of Li-ion batteries (Shen et al., 2020). The DCNN-ETL structure is shown in Figure 2D. Each of the input data group is set to be current, voltage, and charge capacity with 25 segments, whereas the output is capacity. A total of 25,338 groups of samples in the dataset are split to pre-train the *n* DCNN sub-systems and 525 groups of the samples are used to retrain the DCNN-ETL system. The results show that the DCNN-ETL has a higher training accuracy than DCNN, DCNN-TL (DCNN with transfer learning), and DCNN-EL (DCNN with ensemble learning), whereas the training efficiency of DCNN-ETL is much lower than the other machine learning algorithms.

#### Battery lifetime estimation and prediction

The remaining useful lifetime (RUL) of a battery is typically quantified by the time or cycling number when the capacity or SOH decreases to a threshold value. Accurately predicting the RUL is critical for the BMS to adjust its controlling strategy to ensure the performance, safety, and lifetime of a battery. Besides, accurate estimation and prediction of battery RUL is vital in providing guidance for battery reuse or recycling. Regression algorithms are often used to estimate and predict RUL.

Among the application of supervised learning for battery lifetime estimation, the work in (Severson et al., 2019) applied a linear regression data-driven approach to predict the battery cycle life before capacity degradation based on the early cycle discharge data. They created a dataset from 124 Li-ion battery cells cycled under fast charging conditions, used features calculated based on the discharge voltage curve (such as charge time, temperature integral, discharge capacity at cycle 2, etc.) as the input of the regression model, and used the predicted number of cycles as the output of the regression model. Their results showed a test error of 9.1% for the regression setting when only using the first 100 cycles and a test error of 4.9% for the classification setting when only using data from the first 5 cycles. Besides the regression approach, decision tree is used to predict RUL (Zhu et al., 2019b). An optimized version of the CART algorithm is applied for the DT model. It is shown that DT can predict RUL with high accuracy (e.g., 95.2% for feature combination). The work in (Zhou et al., 2020c) shows the application of a *k*-NN regression (kNNR) algorithm to estimate the RUL of Li-ion batteries by utilizing *k*-nearest experimental cells sharing a similar degradation trend. A differential evolution technology is employed to optimize the parameters used for RUL prediction. The best RUL estimation has an error of 2 cycles or a relative error of 0.5%. In another work (Liu and Chen, 2019), a combination of indirect health indicator (HI) and multiple GPR model is applied to predict RUL. Three features are first distilled as HIs from the voltage and current curves of the batteries during the constant current-constant voltage (CCCV) charging process. The GPR model (which is based on Bayesian framework) is optimized with combined kernel functions (linear function, squared exponential, and periodic covariance functions). Then the three normalized HIs and the capacity are set as the input and output of the optimized GPR, respectively, to train the model. It is found that the approach gives high prediction accuracy (the relative error is below 5% for the B5 battery with the starting predicting cycle of 100).

As one of the supervised learning models, SVM (and SVR) is widely used for RUL prediction. For instance, the SVM model is used to classify and predict the RUL of Li-ion batteries (Patil et al., 2015). The SVM classification technology is firstly used to estimate a gross RUL (a total of four classes for different discharge cycles). Then the SVM regression model is used to estimate an accurate RUL. Specifically, features (including capacity, energy of signal, fluctuation index of signal, curvature index of signal, etc.) are distilled from the cycling tests and are imported into the SVM to train the models for RUL prediction. It is shown that the classification-regression approach can improve the calculation efficiency. Besides SVM, the relevance vector machine is also used to predict RUL, such as the work of (Liu et al., 2015) and (Zhou et al., 2016). Specifically, a novel health indicator (the mean voltage falloff (MVF)) is developed to describe the degradation more comprehensively (Zhou et al., 2016). A series of optimized MVFs is used to train the RVM and then predict the MVF pattern starting from a specific starting cycle to predict the RUL. The approach shows high prediction accuracy with an absolute error below five cycles. A hybrid method (fusing the algorithms of unscented Kalman filter (UKF), RVM, and complete ensemble empirical mode decomposition (CEEMD)) is applied to predict the RUL (Chang et al., 2017). The UKF is used to achieve the prognostic result and gain a series of raw error data. Then the CEEMD is used to decompose the error data and obtain the dominant mode (new error data). Finally, the RVM is used to predict the prognostic error to predict the final RUL.

Various ANN-based models are applied for RUL prediction. ANN (an SLFNN model) is used to predict the RUL for Li-ion batteries online (Wu et al., 2016). The input to the SLFNN are eleven points from the CC charging voltage curve selected by the importance sampling (IS) method (Biondini, 2015; Sadowsky, 1990), whereas the output of the SLFNN is the equivalent circle life (ECL). Then the RUL is calculated by subtracting the cycle number when the battery reaches its end of life by the ECL. The data points from 2000 charging/discharging cycles are used to train the SLFNN model. It is shown that the error of RUL prediction is less than 5%. ANN (an SLFNN model) and SVM models are used to estimate the cycle life of lithium polymer batteries (Zhou et al., 2017). The input for the SLFNN and SVM (has a linear kernel function) is the thermal information (normalized de-trend surface temperature acquired from the infrared images taken in experiments) or the electrical information (the current or voltage). The output of the SLFNN and SVM is the cycle number of the battery (or the associated elapse time when the ML model is SVM). Three cells are cycled for 410 cycles and a portion of voltage, current, and temperature datasets of each cell are used to train the ML models. It is shown that with the input being the thermal information, the accuracy of cycle life prediction by the SLFNN and SVM is similar (with the error below 10%), but the SVM needs longer testing time. DNN is used to predict the SOH and RUL of Li-ion batteries (Khumprom and Yodo, 2019). In the work a dropout algorithm is applied to train the DNN (the dropout strategy randomly drops some neurons of the DNN with a fixed probability of *p* (0.25 in the research) during DNN training) to make the DNN thinner and reduce overfitting, which improves the DNN performance (Srivastava et al., 2014). A comparison to other algorithms shows that DNN prediction of SOH has an RMSE (3.427%) lower than that of ANN (4.611%), SVM (4.552%), logistic regression (4.558%), and *k*-NN (5.598%). DNN shows similar accuracy as ANN when predicting RUL (e.g., both have an error of one cycle at the starting point of 120^th^ cycle). DNN and ANN show higher prediction accuracy than that of the SVM (an error of 4 cycles), logistic regression (an error of 9 cycles), and *k*-NN (an error of 19 cycles).

For estimating the RUL, the data can sometimes be complex so that appropriate data preprocessing and feature distilling methods should be employed to improve the training accuracy. In a work of using DNN to predict RUL (Ren et al., 2018), feature extraction from the characteristic curves of batteries (including terminal voltage, output current, temperature, and measured voltage, and current curves during multiple charge cycles) is applied to overcome the unbalanced dataset number. Specifically, an autoencoder neural network is employed to reduce the dimension of the features (15 dimensions) and improve the efficiency of the model. After that, the 15-dimensional features are normalized and set as the input to the DNN, whereas the output of the DNN is RUL. The results show that the DNN combining the data-preprocessing approach has much higher prediction accuracy for RUL than that of the LR, Bayesian regression (BR), and SVM algorithms (the RMSE of DNN, LR, BR and SVM is 6.66%, 12.00%, 11.22%, and 10.66%, respectively). Besides, the strategy of ensemble learning can also be applied during the setup of an ML model to increase the model robustness. For example, an ensemble learning system (ensemble of 200 single random vector functional link (RVFL) network models) is applied to predict the RUL of Li-ion batteries (Gou et al., 2019). The RVFL algorithm randomly chooses the input weights and biases and analytically determines the output weights by simple matrix computations. Thus, the learning speed of RVFL is higher than that of the conventional ANN models. Specifically, the nonlinear autoregressive with exogenous inputs (NARX) structure containing past and present information is applied for the RVFL network to improve the accuracy and stability of the predicting results for RUL. The sequence of history capacity value and the duration of equal charging voltage difference value are input to each of the RVFL network models. The output from each RVFL network is the capacity value after a certain number of cycling. The dataset from three batteries is used to train the ML model. The ensemble learning system shows high accuracy in predicting RUL (e.g., with an RMSE of 0.0184 for the CS 38 battery).

As for the input dataset with complex time-series characteristics, CNN and RNN models are both used to predict RUL. For instance, a CNN-LSTM hybrid neural network is employed (Ma et al., 2019a). The CNN is used to extract useful information, whereas the LSTM is used to predict the unknown sequence of capacity data based on the features extracted by CNN. A segment of the discharging capacity degradation curve is applied to train the hybrid neural network. Specifically, the false nearest neighbors (FNN) algorithm is used to determine the appropriate sliding window size and to determine the size of the input vector (the segment length of the capacity-cycle curve selected for the input). The output is the predicted capacity-cycle curve. The RUL is calculated based on the curve. It is shown that by appropriately selecting the sliding window size, the CNN-LSTM hybrid neural network model can provide a higher prediction accuracy for RUL than using CNN or LSTM alone (e.g., the actual RUL is 56 cycles, whereas the predicted RUL by CNN-LSTM, CNN, and LSTM are 55, 117, and 102 cycles, respectively). In a work of using LSTM to predict RUL (Zhang et al., 2018), a specific span of the experimental cycle-capacity curve of each battery cell is employed to train the LSTM. A total of four cells are used to generate the data. A dropout strategy is used during training to avoid overfitting, whereas Monte Carlo is used to generate RUL prediction uncertainties. A comparison to other ML algorithms, including SVM, particle filter model (PFM), and simple RNN (SimRNN), shows that the LSTM can predict RUL independent of the offline training data and that the LSTM provides much higher prediction accuracy. An LSTM-DNN deep learning model is also applied to predict RUL (Veeraraghavan et al., 2018). The battery capacity decaying pattern obtained from virtual tests (based on a standard proposed by (Wang et al., 2011)) under various C-rates is used to train the deep learning model. The input features for the deep learning model are current, voltage, working temperature of the battery, and the battery capacity at the previous moment. The output is the capacity at the current moment. Similarly, several studies (Liu et al., 2019b; Park et al., 2020; Qu et al., 2019) have used ML algorithms containing LSTM to predict the SOH or the RUL of batteries.

#### Battery fault diagnosis, degradation analysis, and property classifications

An important application of the machine learning approaches is to detect battery defects, as well as to detect and classify the abnormal batteries, in order to ensure the consistency of battery cells. This is beneficial for the BMS system to choose appropriate controlling strategies, which is critical for ensuring the safety and lifetime of batteries.

We first look at unsupervised and supervised learning approaches. In a study (Ortiz et al., 2019) several ML algorithms are applied to classify the unbalance and damage of Ni-MH battery cells, including logistic regression (Dong et al., 2016), *k*-NN, kernel-SVM (KSVM with a Gaussian radial base function kernel (Kim et al., 2012)), Gaussian naive Bayes (GNB), and NN with only one hidden layer. Principal component analysis (PCA) is used to generate 28 PC points showing the cell quality in two classes based on 28 discharging voltage curves. The generated PC points are used to train the ML algorithms. It is found that KSVM and GNB give the best classification performance because their classification curves matches the data well. In another work (Haider et al., 2020), a *K*-shape-based time series hierarchical clustering algorithm is applied to perform defect detection for lead-acid batteries.

The Gaussian process regression is also gaining significant attention. For instance, the work in (Lucu et al., 2020) applied the GPR to construct a calendar capacity loss model to analyze the aging characteristics of Li ion batteries. The model contains a tailored kernel function. Specifically, they set the storage time for which the aging is predicted, the reciprocal temperature corresponding to this storage time, and the SOC level corresponding to this storage time to be the input to the GPR model and set the capacity loss to be the output. Their results showed that the mean-absolute-error of prediction of capacity loss and capacity is 0.31% and 0.53%, respectively, when the model was trained by the dataset acquired from only 18 cells tested at 6 storage conditions.

The deep learning approach is also widely used for detecting and classifying battery cells with abnormal behaviors. A deep belief network model is employed to detect the voltage anomalies of storage batteries (Li et al., 2019). The DBN model consists of a plurality of restricted Boltzmann machine (RBM) (Fischer and Igel, 2012) stacks and a layer of neural network. The model can learn the probability distribution of the input data and use the calculated probability to determine the active state of each node. So, the model has a higher training speed and better training convergence than the traditional back-propagation neural network. The voltage-time and current-time curves after parameter extraction are used to train the DBN (with 15 hidden layers). The input includes 9 parameters such as charge/discharge current, time, temperature, etc., whereas the output is voltage. A comparison with optimization methods of gradient descent (GD) and Levenberg-Marquardt (LM) (Moré, 1978) show that DBN-LM has the highest voltage predicting accuracy (with an MSE of 7.25 × 10^−4^ and an MAE of 0.0105) and training speed (639 iterations) than DBN-GD, BP-LM, and BP-GD. In another work (Zhao et al., 2017), big data analysis methods (including 3*σ* multi-level screening strategy (3*σ*-MSS) and a neural network algorithm) are used to diagnose the fault and defect of batteries. The real-time running data of electric vehicles are used. The 3*σ*-MSS algorithm is based on the Gaussian distribution probability characteristics, which can generate clusters and fault criteria for trouble-free terminal voltages. The 3*σ*-MSS updates the cluster by excluding the samples outside the 3*σ* range. Then an ANN (back-propagation NN with a hidden layer) model is applied to simulate the cell fault distribution in a battery pack. The input to the ANN is the fault frequency matrix acquired by the 3*σ*-MSS. In the work of (Yao et al., 2020), a wavelet-neural network system (containing discrete wavelet transform (DWT) and a general regression neural network (GRNN)) are used to detect the fault of Li-ion batteries for electric vehicles. The GRNN used is a highly parallel radial basis function network, which contains the input layer, pattern layer, summation layer, and output layer. The GRNN has the advantage of faster model building when compared with a traditional ANN. Specifically, the signal values (after denoising by DWT) and the characteristic parameters of the voltage curves (acquired from vibration tests) are used to train the GRNN. The input of the GRNN include voltage, voltage difference, covariance matrix, and variance matrix. The output of the GRNN is the fault degree (quantified by the fault score). The predicting accuracy of the GRNN can be up to 99.675%.

The input dataset (such as a cluster of multiple voltage curves) can sometimes be complex and contains multiple features, which make it difficult for most machine learning algorithms to process and learn. CNN, which has a great capability in mining the underlying features of the dataset, can be employed for clustering the terminal voltage curves of battery cells to diagnose the abnormal battery cells. For instance, CNNs based on two-step time-series clustering (TTSC) and hybrid resampling (HR) are applied to screen the Li-ion batteries (Liu et al., 2018), in order to ensure the consistency of electrochemical characteristics of battery cells and to avoid property discrepancies caused by material variation and fluctuations in manufacturing precision. The TTSC is used for labeling the raw discharge voltage series of the cells (by using the *k*-means algorithm (Huang et al., 2016) to classify whether the cell is inconsistent (labeled as “0”) or not (labeled as “1”)). The hybrid resampling is used for solving the sample imbalance issue (by using the under-sampling algorithm to discard a portion of similar samples in the majority class dataset and by using the SMOTE algorithm (Chawla et al., 2002) to increase the number of minority class dataset). After that, the labeled and number-balanced discharge voltage series are used to train the CNN (a total of 7,205 series for training). The input is the discharge voltage-time curve, whereas the output is the classification result of the label distribution (inconsistent or not). By using the TTSCHR-CNN to screen the testing discharge voltage series of the cells, the inconsistency rate drops by 91.08% compared with the traditional screening method.

**Figure 3:**
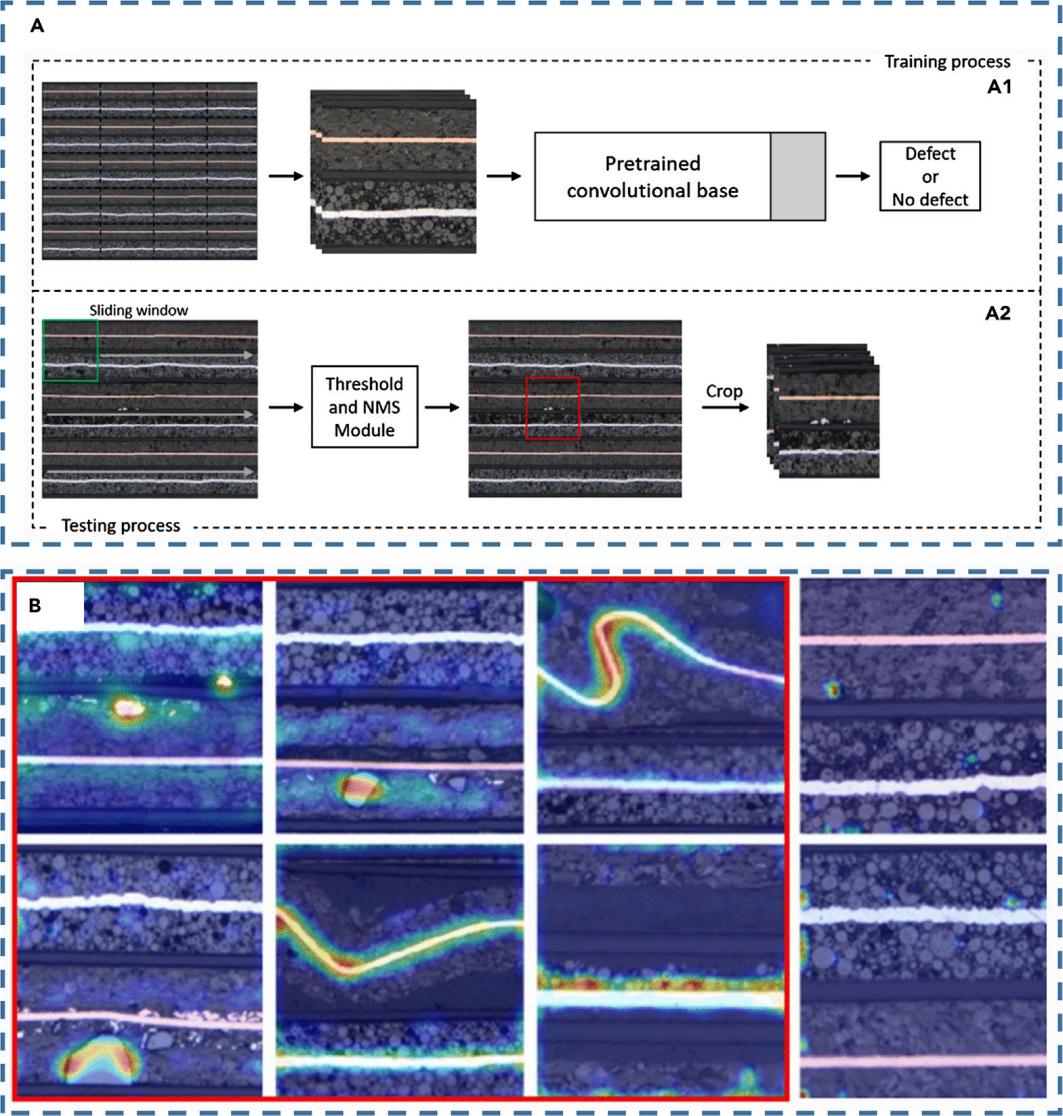
Examples of the application of machine learning for battery fault diagnosis and abnormal detection (A) The application of CNN for detecting the battery electrode microstructural defect based on deep learning computer vision method. Reprinted by permission from Springer Nature (Badmos et al., 2020). Copyright 2020. (A1) Training process for CNN based on the large image of electrode micrograph. (A2) Testing process for CNN. (B) Examples of class heatmap for micrographs with defect (shown in red box) and without defect. Reprinted by permission from Springer Nature (Badmos et al., 2020). Copyright 2020.

The above-mentioned machine learning approaches mainly use the dataset with the array data structure as the input for training the ML models. In some applications, the input to the ML models is in the form of images, such as the snapshots of the battery electrode microstructure. Under this circumstance, the CNN, which is highly capable in distilling the features of images, can be utilized. This borrows the learning process from the field of computer vision and is gaining increasing attention in the energy storage field. The work in (Wang et al., 2019) shows an approach to classify the voltage of fully charged LiPo batteries after a period of usage by applying CNN to learn the images of “battery face.” A Walabot sensor (which is a radio-frequency-based sensor) is used to collect the signals of five types of LiPo batteries with four types of fully charged voltage, to create “battery face” RGB images (the varying image characteristics are caused by the varying electrolyte states). Besides, linear discriminant analysis (LDA) is used to cluster the voltage class. Then the images (input) and the class that the battery voltage belongs to (output) are used to train the CNN model. It is shown that the CNN model has an accuracy of 93.75% (at the 80^th^ epoch) for classifying the battery voltage. It is suggested that the CNN classifying accuracy can be further improved by accessing the raw image of Walabot, increasing the amount of training images, developing parameter optimization algorithms for CNN, and employing appropriate preprocessing methods on the training images. The work in (Badmos et al., 2020) shows the application of CNN to detect the microstructural defect in the electrodes of Li-ion batteries as a result of the manufacturing process to evaluate the battery quality. A total of 2,284 micrographs of the original electrodes are used to train the CNN model. The micrographs are 256 × 256 RGB images as shown in Figure 3. An image with defect is labeled as “class 0,” whereas an image without defect is labeled as “class 1.” The input to the CNN are the images, whereas the output from the CNN is the classification result (defect or not). The models used in the research include traditional CNN models (Baseline model, Sigmoid model, and Softmax model) trained by limited battery data and TL CNN models (pre-trained VGG19 (Simonyan and Zisserman, 2014), inception V3 (Szegedy et al., 2016), and Xception (Chollet, 2017), with a new fully connected layer added at the end of each TL model). It is shown that the CNN can learn meaningful features on its own and identify various defects. Specifically, the TL CNN models show a much higher classification accuracy than the traditional CNN models, because the TL models have previously experienced a larger size of dataset for training. The VGG19 fine-tuned model achieves the best classification performance (with an F1-score of 0.99 for class 0 and 1.00 for class 1) among all the CNN models. In the work of (Ma et al., 2019a, 2019b), CNN is applied to detect the blister defect of the polymer Li-ion battery (PLB). A total of 18,860 PLB sheet images (RGB images with a size of 219 × 219) are used to train the CNN model. The output of the CNN model is the classification result (blister or not). Improved dense blocks are employed to overcome the gradient vanishing problem during CNN training and to increase the training rate. In addition, the flower pollination algorithm (FPA) is used to optimize the parameters in the CNN model during training. It is found that the CNN model gives better classification performance (with an F1-score of 0.988) than the other seven ML algorithms tested (such as NN, SVM, and RCNN).

#### Battery design and optimization

**Figure 4:**
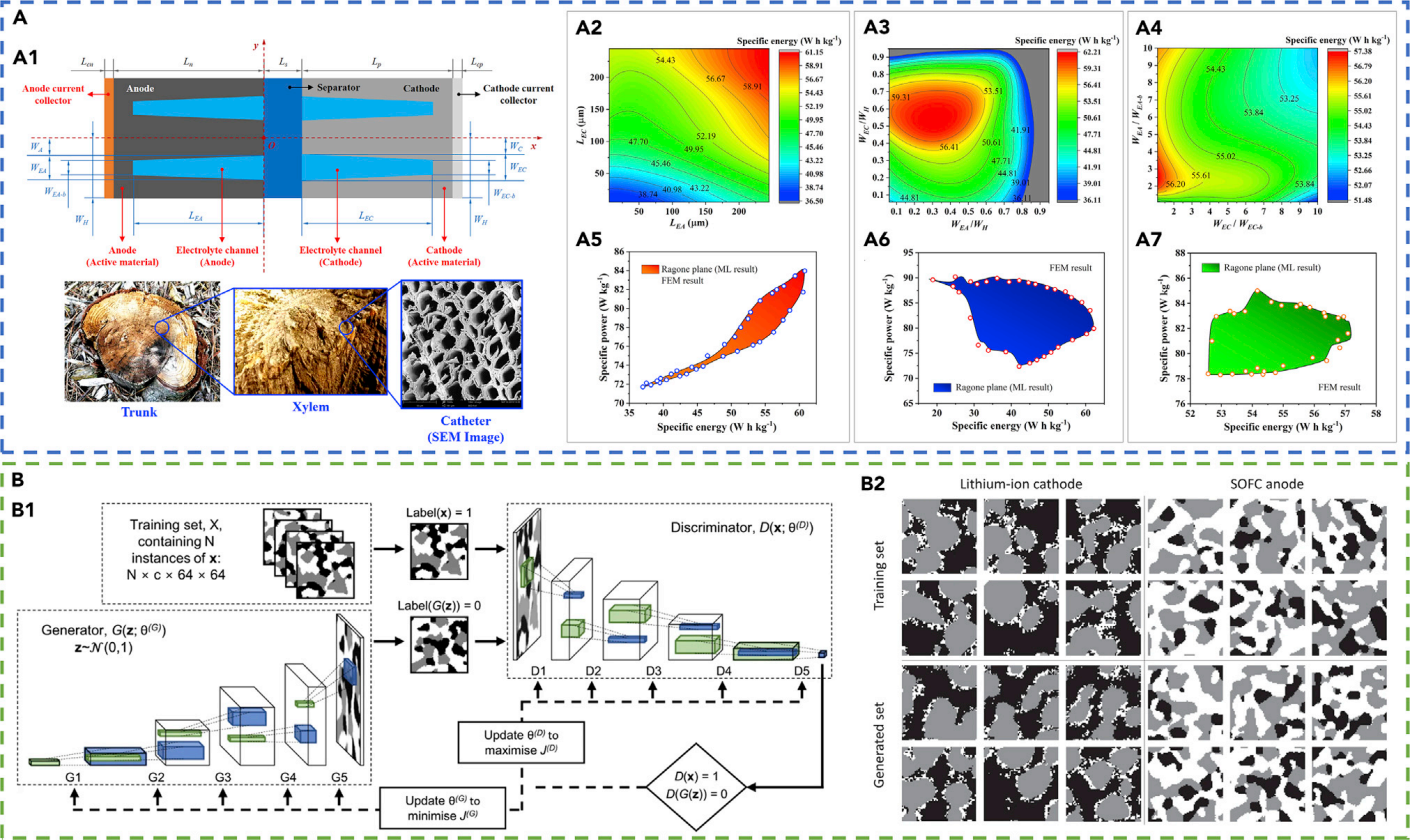
Examples of the application of machine learning for battery design and optimization (A) The application of DNN on optimizing the electrolyte channel geometrical parameters within the electrodes to gain the maximum battery cell performances. Reprinted from (Gao and Lu, 2020). (A1) Model description and geometrical parameters of the electrolyte channels. The design idea is motivated by the microstructure of the trunk, which can transport nutrients quickly through catheters. The input parameters to the DNN are *L*_*EA*_, *L*_*EC*_, *W*_*EA*_/*W*_*H*_, *W*_*EC*_/*W*_*H*_, *W*_*EA*_/*W*_*EA-b*_, and *W*_*EC*_/*W*_*EC-b*_. The output from the DNN are *SE*, *SP*, and *SC*. (A2–A4) Design map of the specific energy generated by the trained DNN. (A5–A7) Ragone planes generated by the trained DNN coupled with MCMC. (B) The application of DC-GAN for the reconstruction of the 3D multi-phase electrode microstructure for the battery and SOFC with periodic boundaries. Reprinted from (Gayon-Lombardo et al., 2020). (B1) The structure of the DC-GAN model. (B2) Results of the reconstruction. The generated and the real microstructures are similar, showing successful reconstruction by the DC-GAN.

In this subsection, we introduce the application of machine learning for battery design and optimization. The work of (Wu et al., 2018) shows the approach of using ANN (SLFNN model) to optimize the performance (specific energy and specific power) of Li-ion battery cells based on cell design parameters (electrode thickness, solid volume ratio, Bruggeman constant, particle radius, electrolyte concentration, and the applied C-rate). A total of 900 groups of data points generated by finite element method (FEM) simulations are used to train and valid the ANN-based model. Sensitivity analysis based on the ANN model identifies which design parameters are important for specific energy or specific power. Battery design maps that satisfy both specific energy and specific power requirements are generated. The work of (Gao and Lu, 2020) demonstrates the development and application of a DNN model to optimize the electrolyte channel design in thick electrodes for fast charging. A total of 20,000 groups of data points are generated by FEM to train, verify, and test the DNN model (which has three hidden layers). The geometrical parameters of the electrolyte channel (Figure 4A1) are set as the input for the DNN model, whereas the output from the DNN is the specific energy (*SE*), specific power (*SP*), and specific capacity (*SC*). The design maps that correlate the geometrical parameters and cell performance (as shown in Figures 4A2–A4) and the Ragone planes that correlate *SE* and *SP* (as shown in Figures 4A5–A7) are successfully generated. The trained DNN shows high accuracy in predicting the *SE*, *SP*, and *SC*, with relative error below 5%. The work also shows using the Markov Chain Monte Carlo (MCMC) algorithm with DNN to optimize the battery performance. The MCMC is based on the gradient descendant method coupled with the self-adjustment strategy (Gao et al., 2019; Ying et al., 2017a, 2017b). The MCMC is contained in the trained DNN model as a continuous regression function. It is shown that a battery cell with the electrolyte channel design can improve the specific energy by 79% compared with the conventional-designed battery cells during fast charging. ANN-based model is also applied to design mesoscale electrode structures (Takagishi et al., 2019). MATLAB is used to construct 2,100 three-dimensional (3D) artificial electrode structures based on the random packed method. These models are then used to generate 2,100 groups of data points acquired by simulations to train and verify an ANN. The input to the ANN are electrode design, property, and manufacturing parameters including the active material volume fraction, particle radius, binder/additives volume fraction, electrolyte conductivity, and the compaction process pressure. The output from the ANN are resistance parameters, including the reaction resistance, the electrolyte resistance, and the diffusion resistance. The trained ANN model (with two hidden layers) can achieve prediction accuracy of *R*^2^ = 0.99. The Bayesian optimization algorithm coupled with the trained ANN-based model is further used to infer the optimized process parameters from the total specific resistance.

For constructing the structure of ESDs, several studies have applied the generative adversarial network. For instance, in (Gayon-Lombardo et al., 2020), deep convolutional GAN (DC-GAN) models are applied to construct 3D cathode microstructures for batteries and 3D anode microstructures for solid oxide fuel cells (SOFC). The structure of the DC-GAN is shown in Figure 4B1. The generator and the discriminator of the DC-GAN both contain five layers. The layer function for the generator is Conv3d, whereas the layer function for the discriminator is ConvTransposed3d. The real dataset for the DC-GAN model are microstructural images. A comparison of the real and the synthetic data (based on morphological parameters, transport properties, and the two-point correlation function) shows that arbitrarily large synthetic microstructural volume and periodic boundaries can be successfully constructed. Examples of the real and generated microstructures for the battery cathode and the SOFC anode are shown in Figure 4B2.

#### Battery modeling and behavior prediction

The machine learning approaches are also used for modeling and predicting the battery behavior, as well as building up battery models. For instance, the SVM algorithm is used to classify the type of EIS models for the EIS spectrum in Li-ion batteries and supercapacitors (Zhu et al., 2019a). Five different types of equivalent circuit EIS models are included. Over 500 EIS spectra are extracted from published papers and the (*Z*(*Re*); -*Z*(*Im*)) points of the EIS spectra are used to train the SVM. Among the total data, 80% is used for training and 20% is used for testing. In the work of (Tang et al., 2018), a model-based ELM is applied to predict the future voltage, power, and surface temperature of batteries based on the given load current information. The model-based ELM uses the temperature model and the equivalent circuit model of a battery to replace the active function of the ELM. The model-based ELM can predict the temperature and power with error limited to ±1.5 °C and 2.5 W, respectively. In another work (Chen et al., 2018), SVM is applied to detect the heat generation mode for Li-ion batteries. The input for the SVM model are the temperature rise and the discharge capacity. The SVM model outputs the classification of the type of heat generation mode (JD mode or RGB mode) based on an optimized hyperplane.

For battery model construction, the stacked denoising autoencoders-extreme learning machine (SDAE-ELM) is applied to build a temperature-dependent model for Li-ion batteries (Li et al., 2019a). A total of 450,000 points of data are used to train the SDAE-ELM model. Specifically, SVR is used for data cleaning, including filling the missing data (current, terminal voltage, or SOC) and correcting the outlier data. The *f*-divergence algorithm is applied to evaluate and even the data distribution for data preprocessing. The SDAE acts as a feature extractor, which is trained (unsupervised) by the preprocessed data. The output of the SDAE serves as the input for ELM. The ELM is used to construct models, which is trained by the raw data. For predicting the terminal voltage, the input to the SDAE-ELM are SOC, current, and temperature. For predicting the SOC (at moment *t*), the input to the SDAE-ELM are the terminal voltage, current, temperature at moment *t*, as well as the SOC at moment (*t*-1). Results show that the temperature-dependent battery model provides terminal voltage estimation with an error less than 2%, whereas the error in SOC estimation is within 3%. In (Li et al., 2019b), a deep belief network-back propagation neural network (DBN-BP) is used to construct a battery model to provide information for the BMS. The DBN-BP model contains a DBN and a layer of BP neural network. The DBN acts as the pre-trainer for the BP network. The experimental data collected from electrical buses are used to train the DBN-BP model. The input for the DBN-BP are current, temperature, and SOC. The output from the DBN-BP is the terminal voltage. A comparison of the voltage prediction accuracy with other ML algorithms including SLFNN, SVM, and ELM shows that DBN-BP gives the highest voltage predicting accuracy (with an MAPE of 2.42%). SVM has higher accuracy (with an MAPE of 2.87%) than ELM (with an MAPE of 3.68%) and SLFNN (with an MAPE of 4.15%) because SVM is more robust and not easily affected by the noise in the data (Jain et al., 2014). Moreover, the Gaussian process is also applied to assist in constructing crack pattern model for LIB with silicon-based anode (Zheng et al., 2020), to assist the design of anode material. Specifically, the data points generated by FEM are applied to train their GP-based surrogate model. The input to the model are the fraction ratio, the Si layer thickness, and the curvature. The output from the model is the Si island area. The results showed that the GP surrogate model has high accuracy in predicting the Si anode performance, and the reliability (quantified by “Cumulative confidence level” (Wang and Wang, 2014)) can be 0.997 when using 20 data points to train the model.

### Application of machine learning for capacitors/supercapacitors

In this subsection, the application of machine learning for capacitors and supercapacitors is introduced. Current studies mostly focus on behavior analysis, performance estimation, RUL prediction, and modeling and design. Specifically, RUL is quantified by the time or cycling number when the capacitance decreases to a threshold value.

For the cyclic voltammetry investigation, an ANN-based approach (an SLFNN model) is applied to model the cyclic voltammetry behavior of supercapacitors with a MnO_2_ electrode (Dongale et al., 2015). The potential and current density are used as the input parameters for the SLFNN, whereas the output is the cyclic voltammetry performance. Experimental data are used to train the SLFNN model based on five types of supercapacitors under applied DC voltages of 0–1 V for 20 cycles. The results show that the trained SLFNN model has good performance with a low error (below 2%) in predicting the specific capacitance when compared with experimental results. Similarly, ANN (an SLFNN model) is also used in (Lokhande, 2020) to model the cyclic voltammetry for supercapacitors with Ni(OH)_2_ electrodes.

DNN is applied to predict the capacitance for carbon-based supercapacitors (Zhu et al., 2018). A total of 681 sets of data are collected from more than 300 publications to train the DNN model (with two hidden layers). The input data to the DNN are the specific surface area, the pore size, the *I*_*D*_/*I*_*G*_, the N-doping, and the voltage window. The output from the DNN is the capacitance. The DNN shows a high training accuracy (*R*^*2*^ = 0.91) and out-of-sample accuracy when comparing the predicted capacitance to the real capacitance.

**Figure 5:**
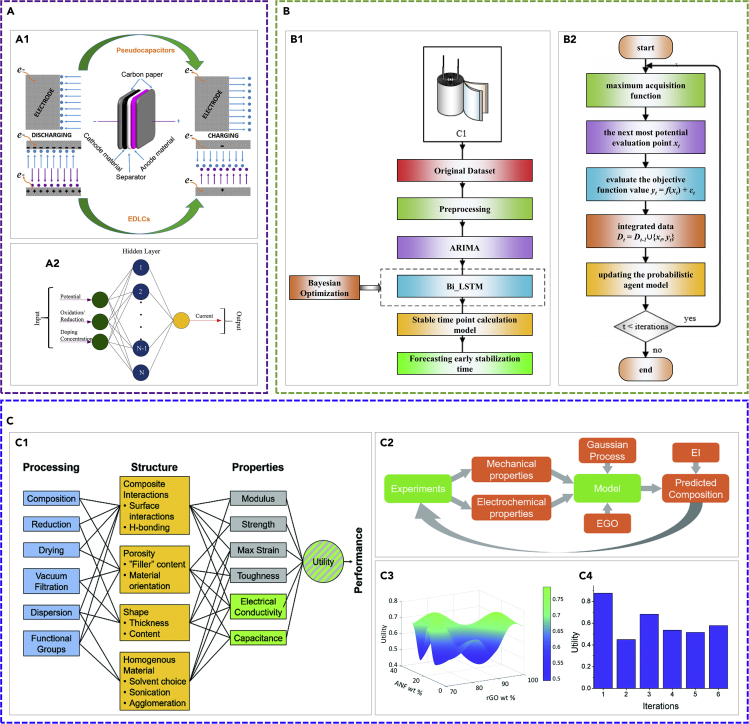
Examples of the application of machine learning for capacitors and supercapacitors (A) The application of ML on the cyclic voltammetry behavior modeling of Co-CeO_2_/rGO nanocomposite supercapacitors. Reprinted with permission from (Parwaiz et al., 2018). Copyright 2018 American Chemical Society. (A1) The illustration of the structure of supercapacitors and two modes of charging storage mechanism. (A2) The setup of the ANN for modeling the cyclic voltammetry behavior for Co-CeO_2_/rGO nanocomposite supercapacitors. The ANN contains an input layer, a hidden layer, and an output layer. (B) A schematic of the electronic capacitor early stabilization time prediction through (B1) the hybrid ARIMA-Bi_LSTM model and (B2) a schematic of the Bayesian optimization algorithm. Reprinted from (Wang et al., 2020a, 2020b). (C) The application of machine learning for designing multifunctional supercapacitor electrodes. Reproduced from (Patel et al., 2019) with permission from The Royal Society of Chemistry. (C1) The correlation between processing, structure, properties (including mechanical properties (gray boxes) and electrochemical properties (green boxes)), and the performance of a composite electrode composed of reduced graphene oxide, Aramid nanofibers, and carbon nanotubes. (C2) A schematic of the interactions between experiments and computations (including the Gaussian process machine learning computation and global optimization). (C3) The predicted utility (after 6 iterations). (C4) The predicted utility of the samples with composition at each iteration.

Several studies have compared different types of ML algorithms in predicting the performance of supercapactors. For instance, the work in (Zhou et al., 2020a) compares several machine learning algorithms (generalized LR (GLR), SPV, RF, and ANN) in correlating the structural features with the capacitance of the carbon-based supercapacitor. The input parameters for these ML algorithms are the surface area of micropore and mesopore and the scan rate. The output are the specific capacitance and power density. A total of 70 groups of data points are used for training the ML algorithms. The results show that ANN has the highest accuracy for predicting the capacitance, wheres GLR has the lowest accuracy. The performance of ANN and RF for estimating the current of Co-CeO_2_/rGO nanocomposite supercapacitors are compared in (Parwaiz et al., 2018). The training datasets are obtained from experiments. The input for the ML algorithms are potential, oxidation/reduction, and doping concentration, whereas the output is current (the set-up of the ANN (an SLFNN) is illustrated in Figure 5A2). It is found that ANN performs better than RF. In (Su et al., 2019), the performance of ML algorithms including M5 model tree (M5P) (Breiman et al., 1984), M5 rule (M5R), multilayer perception (MLP, a widely used ANN model), SVM, and LR are compared for predicting the capacity of supercapacitors based on the properties of 13 types of electrolytes. The diameter, dipole moments, viscosities, boiling temperature, and dielectric constant of the electrolytes are used as the input for the ML algorithms. It is shown that M5P gives higher prediction accuracy than M5R and MLP due to the simple rule, and LR gives the lowest prediction accuracy. The solvent molecular size and dielectric constant are found to significantly affect the capacity.

Besides the above-mentioned ANN-based model, RNN is also applied to estimate the time-series behaviors for capacitors. For instance, a hybrid ML algorithm of autoregressive integrated moving average, bidirect LSTM RNN, and Bayesian optimization (ARIMA-Bi_LSTM) is applied to predict the early stabilization time for electric capacitors (Wang et al., 2020b). The architecture of the hybrid algorithm is shown in Figure 5B. The ARIMA algorithm is used to predict the linear part of the electrolytic capacitor performance. The Bi_LSTM is used to predict the nonlinear part for its ability of integrating the historical and future information of time series. In the meanwhile, the Bayesian optimization algorithm is used to choose the parameters for the hybrid ML algorithm. The electrochemical impedance spectrum (73 data points of each spectrum) of eight capacitors based on three electrical stress conditions are used to train the hybrid ML algorithm after data preprocessing. Specifically, the input for the Bi_LSTM are the residuals predicted by ARIMA. The output is capacitance. It is found that the hybrid ARIMA-Bi_LSTM algorithm gives the highest prediction accuracy compared with single Bi_LSTM, LSTM, DBN, Elman, and ARIMA (DBN and Elman have the same architecture as Bi_LSTM). However, the hybrid ARIMA-Bi_LSTM algorithm has the lowest running efficiency. It is also a challenge for the hybrid algorithm to remain high prediction accuracy when dealing with more complex systems because more parameters and more complex environment will be involved.

The machine learning algorithms are also applied for supercapacitor's life prediction. For instance, model-extreme learning machine (MELM) is used to predict the RUL for supercapacitors (Zhou et al., 2019). The supercapacitor is modeled by a hybrid degradation mechanism model (equivalent circuit model) coupling the effect of temperature, discharge depth, charge/discharge voltage, charge/discharge current, and vibration. Specifically, the equivalent circuit model is coupled to the sub-models of MELM. The weights of the hidden layers are modified to identify the model parameters. The trained MELM can accurately predict the equivalent series resistance and the capacitance of supercapacitors (the normalized RMSE for the equivalent series resistance and capacitance prediction is below 0.8% and 0.05% within 46,000 h interval, respectively). The running efficiency is high because the electrochemical theory is avoided. The work in (Ren et al., 2020) applied an ANN model to predict the cycle life of supercapacitors. Specifically, they applied a dataset generated from 66 supercapacitors to train their ANN model. The results showed that because it does not strongly depend on the data distribution and the correlation between variables and features, the ANN can have a higher prediction accuracy than the PCA and LR algorithms. Besides, recurrent neural network (the LSTM algorithm) is also applied to predict the RUL of supercapacitors (Zhou et al., 2019, 2020d). The values (e.g., temperature and voltage) at the current moment as well as the capacity and the unit state at the previous moment are set as the input for the LSTM. The output of the LSTM is the capacity and the unit state at the current moment. A dropout algorithm is used to prevent overfitting during the RNN training by randomly selecting part of the RNN units to set the output to be 0 during each iteration adjustment. In (Zhou et al., 2019), a dropout probability of 0.6 and the Adam optimizer are used to optimize the weights. An overall RMSE of 0.0261 is achieved (RUL prediction for the SC1 supercapacitor), which is much lower than the overall RMSE without using the dropout algorithm (0.0578). In (Zhou et al., 2020d), a hybrid genetic algorithm (HGA) is used to optimize the dropout probability. It is found that HGA-LSTM can achieve a much lower overall RMSE (e.g., 0.0161) than the overall RSME without using HGA (e.g., 0.0408) for the same RUL prediction.

Machine learning is also used for the modeling and design of supercapacitors. For instance, two ANN-based models (SLFNN models) are applied to learn the supercapacitor charge and discharge voltage curves respectively, to predict the charge-discharge voltage curves for new supercapacitors (Pozo et al., 2018). The four case studies show that the average error of the voltage predicted by ML is much lower (e.g., RMSE and MSE of 0.052 and 0.005, respectively, for charging) when compared with the voltage predicted by electro-mathematical models (e.g., RMSE and MSE of 0.099 and 0.017, respectively, for charging). In (Patel et al., 2019), functional analysis and machine learning are applied for designing multifunction supercapacitor electrodes. The mechanical properties (Young's modulus, strength, maximum strain, toughness) and the electrochemical properties (electrical conductivity, capacitance) are correlated by a utility function (Figure 5C1). An ML algorithm of Gaussian process regression is applied to model the initial experimental data. The expected improvement (EI) acquisition method is used to predict the next best electrode composition to provide feedback for new experimental designs (Figures 5C2–C4). It is shown that by utilizing functional analysis and ML, the overall utility, Young's modulus, and strength are improved by 5.5%, 78.8%, and 34.0%, respectively, when compared with those of the best initial experimental composition.

### Application of machine learning for fuel cells

For fuel cells, the application of machine learning has mainly focused on RUL prediction, degradation diagnosis and analysis, status and performance estimation, as well as fuel cell modeling, simulation, and design optimization. Specifically, the RUL of a fuel cell is typically quantified by the time when its voltage decreases to a threshold value.

**Figure 6:**
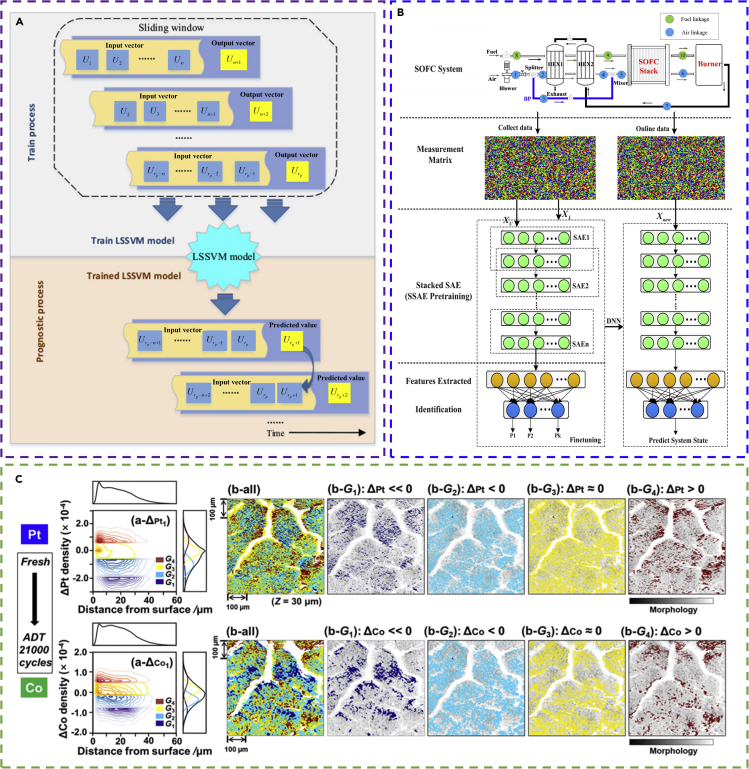
Examples of the application of machine learning for fuel cells (A) The frame of RUL prediction for PEMFC based on the LSSVM model. Reprinted from (Cheng et al., 2018). (B) The structure of the SSAE deep learning model for the fault diagnosis of SOFC. Reprinted from (Zhang et al., 2019b). The SSAE deep learning model comprises multiple SAE networks. The SSAE is firstly pretrained layer-wise, unsupervised, based on the unlabeled dataset *X*_*U*_ to obtain initial parameters. Then the SSAE is fine-tuned based on the labeled dataset *X*_*L*_ to enable essential feature mining and diagnosis. After the training of SSAE, the real-time data *X*_*new*_ collected from the SOFC can be imported into the SSAE for fault diagnosis. (C) An example showing the application of unsupervised learning in data mining of the image data to extract correlations between the structural parameters. Reprinted with permission from (Tan et al., 2019). Copyright 2019 American Chemical Society (a-ΔPt_1_) and (a-ΔCo_1_) denotes the Pearson plot of the relationship between the distance to the surface and the ΔPt (density difference of Pt) or the ΔCo (density difference of Co), respectively, after 21,000 cycles. *G*1–*G*4 denote the four components. The (b-all) and (b-*G*1), (b-*G*2), (b*-G*3), and (b-*G*4) denote the cross-sectional image of the component distributions overlaid on the morphology images.

For the prediction of fuel cell RUL, the least square SVM and the regularized particle filter (LSSVM-RPF) are applied to estimate the RUL of the proton exchange membrane fuel cell (PEMFC) (Cheng et al., 2018). The LSSVM (with the polynomial kernel) is used to forecast the voltage changing pattern, whereas the RPF is applied to realize the uncertainty characteristics of the prognosis result. Experimentally measured voltage data after smooth preprocessing (a total of 1,155 data points) are used to train the ML model. The input vector to the LSSVM is the voltage within the sliding window (with a width of 300), as illustrated in Figure 6A. The output is the voltage outside the sliding window. The LSSVM-RPF approach can achieve high accuracy in prognosis (e.g., the RMSE of the LSSVM-RPF prognostic result is 0.0072 when the prediction start time is at 400 h). The deep learning approaches are also applied for predicting RUL for fuel cells. In (Liu et al., 2019a), the LSTM model is used to predict the RUL for PEMFC. The voltage sequence data (after preprocessing and normalization) are the input to the LSTM, whereas the output is the predicted voltage sequence. The LSTM gives prediction accuracy of 99.23%. In another work (Ma et al., 2020), a hybrid method containing the LSTM algorithm is used for RUL prediction. The voltage curves of five cells from the PEMFC aging experiment provided by (Morando et al., 2017) are applied to train the LSTM model. The input is the normalized voltage data. The output from the LSTM is the normalized RUL prediction. The average relative prediction accuracy is above 0.92.

For fuel cell degradation diagnosis and analysis, RC is used to diagnose the fault type for PEMFC based on the voltage signal (Zheng et al., 2017), including low cathode stoichiometric ratio, defective cooling system, CO poisoning, and continuous aging. The trained model gives high accuracy in classifying the status of PEMFC (e.g., 99.88% for offline training). In (Zhang et al., 2019b), a DNN approach based on the stacked sparse autoencoder (SSAE) is used to diagnose the fault of solid oxide fuel cell. SOFC data generated by MATLAB Simulink are used to train the ML model. The DNN is firstly pretrained by the unlabeled data and then fine-tuned by the labeled data to form a mapping between the original sensor variables (a total of sixteen variables such as the air flow, fuel flow, and power) and the type of system state (a total of seven types such as air leakage and fuel leakage). Specifically, the DNN contains a multi-layer sparse autoencoder (SAE) (Figure 6B). The output of the encoding layer of an SAE acts as the input to the next SAE. The *K*-binary classifiers act as the output of the entire SSAE. The trained SSAE model can diagnose the fault with an accuracy and an F1 score of 79.94% and 89.62%, respectively, when the training sample size is 80,000. In (Tan et al., 2019), a catalyst degradation mechanism is revealed at the cathode of a membrane electrode assembly of practical polymer fuel cells with an infographic approach. Operando spectroimaging is used to produce 3D images of the morphology, Pt and Co distribution, Co/Pt ratio, and Pt valence state. An unsupervised ML approach (a maximizing Bayesian information criterion (BIC) algorithm) for data mining is used to extract correlations between the visualized parameters and the catalyst degradation (Figure 6C). The results show that the infographic approach (which combines 3D chemical imaging and unsupervised learning) is promising to reveal the intrinsic events of practical materials and devices.

For estimation and prediction of fuel cell status and performance, DNN containing convolutional layers is used to estimate the water coverage ratio for PEMFC (Mehnatkesh et al., 2020). A total of 32 images with a shape of 176 × 176×3 is used as input to train the DNN model. Specifically, the structure of the DNN model is optimized by genetic algorithm, which contains four convolutional hidden layers and two dense layers. The output from the DNN is the amount of water in the fuel cell (divided by six classes). The testing results show that the trained DNN has 94.23% accuracy in identifying the water coverage ratio. In (Cai et al., 2019), six ML algorithms (logistic regression multiclass, RF, scalable tree boosting system, neural network, KNN, and SVM with radial kernel) are used to predict the feed substrate type (including acetate, carbohydrates, and wastewater) for the microbial fuel cell (MFC) based on genomic data. In (Lesnik and Liu, 2017), an ANN-based model (Bayesian interaction network) is used to predict the performance of MFC. The input to the ANN is the wastewater characteristics, whereas the output from the ANN are the performance parameters. The results show that the average percentage error of the ANN-predicted Columbic efficiency and chemical oxygen demand removal rate are 1.77 ± 0.57% and 4.07 ± 1.06%, respectively. The average error of the predicted power density is 16.01 ± 4.35%, which can be further improved to within 5.76 ± 3.16%.

Machine learning is also used for the modeling, simulation, and design optimization of fuel cells, which is mostly embodied in constructing regression models between the input and output variables and further using the regression models in specific optimization algorithms. For instance, the GPR is applied to capture the nonlinear relationship between the operating conditions and the output voltage of MFCs (He and Ma, 2016). The GPR model they used contains a squared exponential covariance function (kernel function), and with four operation conditions (such as the flow rate and concentration of acetate in the anode compartment, etc.) to be the input to the GPR model, and with the voltage to be the output. An online learning strategy was also applied to recursively update the hyperparameters in the GPR model. SVR is used to model the electric conductivity of SOFC anode (Tang and Huang, 2019). The input to the SVR model is the operating temperature and the copper content. The output from the SVR model is the electrical conductivity. The trained SVR is shown to have high modeling accuracy based on the testing results (e.g., a mean absolute percentage error of 1.05%). In (Tsompanas et al., 2019), an ANN model is applied to simulate the polarization curve of MFC with different separator membranes materials and electrode configurations. A total of 184 samples from polarization tests are used to train the ANN model. The input are the load resistance, cylinder material, electrode location, and cathode size. The output from the ANN is the output voltage. The correlation coefficient between the ground-truth value and the ANN prediction value is 0.99662, indicating that the trained ANN has high accuracy. In (Wang et al., 2020a), SVR (with RBF kernel) is used to construct a surrogate model for the PEMFC. The surrogate model quantifies the relationship between the input variables (the current density, Pt loading, Pt percentage, and IC ratio of the anode and cathode catalyst layers) and the output variables (the output voltage). A total of 50 data points is used to train the SVR model. It is shown that the trained SVR has a high accuracy (e.g., with a mean percentage error of 3.3375% according to the test results). Then the surrogate model is coupled into the genetic algorithm to optimize the composition of the catalyst layer to achieve the maximum power density. In (Ghasemi et al., 2020), an adaptive neuro-fuzzy inference system (ANFIS) model is constructed to quantify the relationship between the input variables (including the degree of sulfonation, Pt and aeration rate) and output variables (the power density and the chemical oxygen demand removal). The trained ANFIS model is coupled to the particle-swamp-optimization (PSO) algorithm to maximize the power density and chemical oxygen demand removal.

### Application of machine learning for other types of ESD

In this subsection, the application of machine learning in other types of ESD is introduced. For the modeling of flow battery, DNN is used to assist in understanding the relationship between the pore-scale electrode structure reaction and device-scale electrochemical reaction uniformity inside a redox flow battery (Bao et al., 2020). The datasets from 128 pore-scale simulations are used to train and validate the DNN model which has four hidden layers. The input to the DNN are the inlet flow velocity, the electric current density, and the inlet *V*^5+^ concentration. The output from the DNN are the normalized standard deviation of the surface reaction rate (*σ*_*r*_) and the full cumulative distribution function (CDF) of the surface reaction rate. The trained DNN can predict *σ*_*r*_ and CDF with high accuracy (the RMSE of the predicted *σ*_*r*_ and CDF are 1.8 × 10^−3^ and 5 × 10^−5^, respectively).

Machine learning is also used for the modeling of flywheel. For instance, ELM is used to predict the suspension force in an axial split-phase bearingless flywheel (ASPBF) machine (Zhu et al., 2019c). The ELM model is trained by 625 groups of data points acquired by finite element simulations after PCA pretreatment to reduce the computational dimension of input data to ensure data integrity. Specifically, the weights of the ELM and the biases of the hidden layer are optimized by the differential evolution algorithm to avoid the blindness of artificial selection. The input to the ELM model are the radial electricity *x* and *y*, the suspension winding current, and the rotor position. The output from the ELM is the suspension force. The optimized ELM gives a much higher prediction accuracy (RMSE of 4.2907) than the conventional ELM and the PCA-ELM (RMSE of 12.5643 and 11.0689, respectively).

## Approaches and applications of machine learning for ESSs

We introduce three types of commonly used ESS, including the battery energy storage system, the hybrid energy storage system, and the grid and microgrid system containing energy storage modules. The problems that machine learning mainly focuses on include the estimation and prediction of the ESS status, the design of the ESS parameters, as well as the optimization of the controlling strategies to achieve the maximum ESS performance. In addition, we will briefly discuss the application of machine learning in the pump-storage system and in the thermal ESS.

### Application of machine learning for the battery energy storage system

For the application of deep learning to the battery energy storage system (BESS), multi-layer perception neural networks and regression tree algorithms are applied to predict the battery energy consumption in electric vehicles (Foiadelli et al., 2018). The prediction is based on features such as temperature, distance, time in traffic, average speed, and range. It is shown that the RT algorithm gives higher prediction accuracy on the energy consumption (with a R^2^ value of 0.995) than the MLP neural network algorithm (with a R^2^ value of 0.965).

Reinforcement learning is widely used for the management of BESS. For instance, a reinforcement learning (based on a model-free *Q*-learning algorithm) approach is applied to control the energy flow in a solar microgrid containing a PV source, battery bank, destination unit, and load (Kofinas et al., 2018b). The environment (the microgrid system) sends the state variables (battery SOC, power balance, the percentage of potable water into the tank) and the fuzzy rewards (a quantification of the battery SOC, the amount of the water into the tank, and the coverage of the energy demand) to the agent. The agent sends the action (charging/discharging the battery and operating/stopping the desalination unit) back to the environment. In (Mbuwir et al., 2018), reinforcement learning (based on a fitted *Q*-iteration algorithm) is used for optimizing the amount of energy from each energy carrier of a multi-carrier energy system (MCES) to supply all the demand. The RL environment sends the states (battery SOC, time and load component, photovoltaic production, and pricing) and rewards (quantification of the cost incurred or revenue) to the RL agent (the decision maker). The RL agent sends the action (battery scheduling command including charging, idling or discharging the battery) back to the RL environment. In (Bui et al., 2019), reinforcement learning (based on a *Q*-learning based algorithm) is applied for managing the operation of a microgrid coupled with a community battery energy storage system (CBESS). The environment (including the microgrid, the communication and electric power network, etc.) sends the states (time interval, CBESS SOC, and the market price information) and the rewards (a quantification of price based on the chosen action) to the learning agent (the CBESS). The learning agent sends the action (determined by the epsilon-greedy policy) back to the environment.

For the application of deep reinforcement learning in BESS management, a model-free deep reinforcement learning approach (based on the deep deterministic politic gradient (DDPG) algorithm) is used for the energy management system of the plug-in hybrid-electrical bus (PHEB) to optimize the energy split among the modules (Wu et al., 2019). For the RL system, the environment (consists of the PHEB, the power source, and the driving road network) sends the states (such as PHEB speed, acceleration, and battery SOC) and the rewards (the sum cost of the fuel and the electricity consumption) to the agent. The agent is a structured control net (SCN) with input of current state and output of the action value (from the actor) and the *Q* value (from the critic). The SCN is trained and tested by the dataset from 66 to 44 driving cycles. The agent sends the action (the engine torque, rational speed, and the traction motor torque) to the environment. In another work (Chaoui et al., 2019), a deep reinforcement learning approach is applied for the resource allocation scheme of electric vehicles to equilibrate the SOC of all batteries, extend their lifetime, and reduce the maintenance frequency. The environment sends the states (the SOC of each unit) and the rewards (a cost function for quantifying the deviation between the SOC of all units) to the agent, whereas the agent sends the action (a parameter governing the voltage) back to the environment to process the control.

### Application of machine learning for the hybrid energy storage system

The hybrid energy storage system (HESS) contains more than one of the following units, including battery, capacitor (or supercapacitor), and fuel cell. For supervised learning, an SVM load predictive energy management system is applied for a hybrid ESS (including modules of batteries and supercapacitors) in a solar energy system to manage the energy flow within the power source, the ESS, and the load (Chia et al., 2015). Five load profiles (current-time curves) are used to train the SVM. The time-series curves are predicted by *K*-step watching ahead based on one-step watching ahead (Cheng et al., 2006). The input vector to the SVM contains every three previous load current values. The output label from the SVM that links each of the input vectors is the seventh point in the advanced load current value. The performance of the SVM coupled with different kernels (including linear, polynomial, radial base function, and sigmoid) are compared. It is found that the SVM with the polynomial kernel gives the highest load prediction accuracy and speed. The SVM load prediction approach can help improve the system efficiency and reduce the component cost.

For the application of deep learning for HESS management, DNN is used to manage the hybrid energy system of electric vehicles (including battery modules, supercapacitor, motor, etc.) by controlling the flow of energy among the modules (Alaoui, 2019), to achieve the maximum module efficiency. Specifically, a total of 16 trips are used to train the DNN, which has two hidden layers. The input to the DNN are the power demand (such as the actual speed of the vehicle, terrain topography, etc.), the battery pack SOC, and the supercapacitor SOC. The output of the DNN are the power input/output flow of the battery and the supercapacitor. It is shown that the system can save energy up to 2.68%.

For the application of reinforcement learning for HESS management, an RL-based (based on *Q*-learning algorithm) approach is used to manage a hybrid energy storage system (containing the battery pack, ultracapacitor pack, and controllers) in hybrid electric vehicles in order to minimize the energy loss (Xiong et al., 2018). The environment (which is the HESS) sends the states (the diffusion voltage and the SOC of the battery pack, the state of voltage of the supercapacitor pack) and the rewards (the total energy loss) to the agent (the RL controller), whereas the agent sends the action (the load current of the battery pack) back to the environment. It is found that the RL-based energy management strategy can reduce the relative total energy loss by 16.8% and optimize the system efficiency. In (Reddy et al., 2019), a reinforcement learning (based on a *Q*-learning algorithm) approach is applied for the power and energy management system of fuel cell hybrid electric vehicles (containing fuel cells and batteries) to minimize the variance of battery SOC so as to improve the battery lifetime and the power system efficiency. In the RL framework, the state variables are the battery SOC, the fuel cell power delivery, and the battery power delivery or absorption. The action is a change in the fuel cell power. The reward is the quantification of the relationship between the battery SOC and its average value. In (Sun et al., 2020), reinforcement learning (based on a *Q*-learning algorithm) based the hierarchical energy management strategy is applied for fuel cell hybrid electric vehicles (containing fuel cells, batteries, and supercapacitors) for lowing the computational cost and optimizing the fuel cell efficiency and the economy of energy consumption. The state variables in the RL include the SOC of the battery and the fuel cell, the power demand, and the voltage. The action in the RL includes the power of the fuel cell and the battery. The rewards in the RL are related to hydrogen consumption and the variance in battery SOC.

Deep reinforcement learning is also applied for the management of HESS. For instance, deep reinforcement learning-based energy management strategy is applied for the supercapacitor ESS that is used in urban rail transit (Yang et al., 2020b). The environment (the traction power system) sends the states (the operation states of the train, the supercapacitor SOC, the rectifier current, and voltage of substations) and the rewards (the increment of energy-saving and voltage stabilization rate in each time step) to the agent (the supercapacitor energy management system). The agent sends the action (the charge and discharge voltage threshold) back to the environment. The action-value *Q* is generated by a DNN model with three hidden layers. The input of the DNN is the normalized state vector (with 19 components). The output of the DNN is the action-value *Q* for each action. It is shown that the deep RL-based energy management strategy provides 48.8% improvement in energy-saving and 17.6% improvement in voltage-stabilizing rate. These are close to the global optimization values.

### Application of machine learning for the grid and microgrid

For the application of DNN in microgrid management, deep learning adaptive dynamic programming is used to assist the energy management for microgrids (containing energy storage modules) for reducing the operational cost and improving the utilization efficiency of renewable energy (Wu and Wang, 2018). The structure includes a dynamic system, a critic, and an action network (these networks are modeled by DNN models with four hidden layers). For the action network, the input is the state of the controlled object (the amount of distributed energy in the microgrid, the gas and water power, and the active power of the energy storage system). The output is the control strategy (the amount of power that has to be purchased). For the critic network, the input is the state of the controlled object, whereas the output is the cost function.

Reinforcement learning and deep reinforcement learning are increasingly being used for the management of grids and microgrids. For instance, a multi-agent system (MAS) with reinforcement learning (with a fuzzy *Q*-learning method for each agent) is applied to manage the energy of a stand-alone microgrid (Kofinas et al., 2018a). The microgrid includes a power production unit (a photovoltaic source, a fuel cell, and a diesel generator), a power consumption unit (an electrolyzer unit, a desalination plant, and a variable electrical load), and a power storage unit (a battery bank). The MAS, which represents the microgrid, contains five RL agents, which are the desalination agent, battery agent, electrolyzer agent, fuel cell agent, and diesel generator agent. The environment is the microgrid without rigorous modeling (a model-free environment). The state of the environment includes the battery bank SOC, H_2_ demand, electrical load demand, etc. The action from the agents is the percentage of their generation or consumption power or power change. The reward for each agent is the operational strategy of each agent (such as the power produced by the fuel cell or diesel engine).

**Figure 7:**
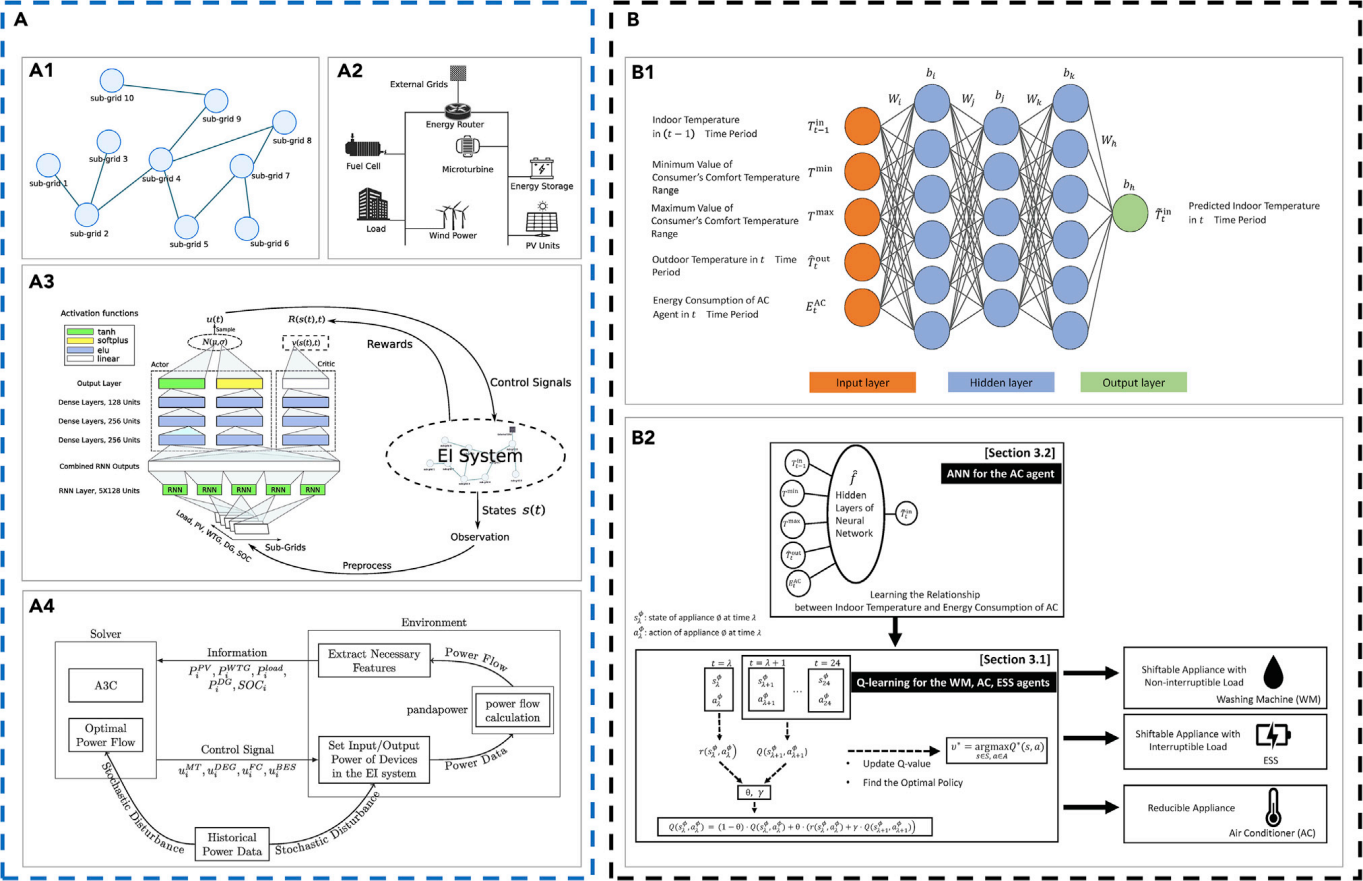
Examples of the application of machine learning for grid and microgrid containing energy storage units (A) The application of deep RL (based on actor-critic algorithm) for optimizing the energy management strategies of an energy internet (EI) system. Reprinted from (Hua et al., 2019). (A1) The topology of the EI, which is connected by multiple sub-grids. (A2) The typical architecture of a sub-grid of the EI, which consists energy storage units, fuel cell, load, etc. (A3) The architecture of the actor-critic network. (A4) A schematic of the simulation process, which reflects the interactions between the environment and the agent. (B) The application of RL (based on the *Q*-learning algorithm coupled with a DNN model) for energy management of a home energy management system (HEMS). Reprinted from (Lee and Choi, 2019). (B1) The architecture of the DNN model, which acts as the air conditioner agent for the RL system. (B2) The overall structure of the RL system for the HEMS coupled with DNN.

In (Hua et al., 2019), optimal control strategies (via deep RL approaches) are used for managing and optimizing the energy internet (EI) system (Figure 7A1), which is interconnected by several sub-systems (containing energy generation, transportation, storage devise, and loads, as shown in Figure 7A2). The asynchronous advantage actor-critic RL algorithm (Figure 7A3) is used to solve the optimal control problem (Figure 7A4). The EI system (observation system, acting as the environment) interacts with the RNN-based actor-critic networks (acting as the agent). The state variables (including the SOC, the power of photovoltaic, wind turbine generator, and diesel generator at each moment) from the EI system act as the input to the actor-critic network. The control signal (acting as action) is exported from the actor-critic network. In (Lee and Choi, 2019), a reinforcement learning approach (which is a model-free *Q*-learning algorithm) and a DNN model are used to manage the energy consumption schedule of a home energy management system (HEMS, which contains an air conditioner (AC), a washing machine (WM), and an energy storage system (ESS); the three modules act as the agents in RL). The DNN (Figure 7B1) is used to predict the indoor temperature and to assist the *Q*-learning to correlate the indoor temperature and energy consumption (Figure 7B2). For the RL, the states of the environment are the energy consumption by the WM, AC, and the state of energy (SOE) of the ESS. The action of the agents includes the binary action of the WM, the energy consumption of the AC, and the amount of charging/discharging energy of the ESS. The rewards for the agents are quantified by the electricity cost and the operational parameters of the agents, which can be affected by the outdoor or indoor temperature (as predicted by the trained DNN). It is shown that the energy bill can be reduced by 14%. Besides, a hierarchical deep reinforcement learning approach (based on the actor-critic algorithm) is proposed for scheduling the energy consumption of smart home applications and distributed energy resources (Lee and Choi, 2020).

### Application of machine learning for other types of ESS

Machine learning is applied in the modeling and controlling of the pumped-storage system. For instance, LSTM-based ML is applied to identify the dynamic model of the pumped-storage unit (PSU, which is composed of a servo-mechanism water diversion system, pump-turbine, generator-motor, and controller) (Feng, 2019). The identified model is further used to optimize the controller in the PSU. Specifically, a total of 2000 linear-normalized data points acquired by MATLAB simulations (pump-turbine model based on the B-spline surface) are used to train the LSTM-based model. The testing result shows that the LSTM-based model has high accuracy (with an MSE that is 17.1%, 54.9%, and 62.2% lower than that of autoregressive model with exogenous variables (ARX), SVM, and feedforward neural network with two hidden layers, respectively). The PSO optimization algorithm coupled with the trained LSTM-based model are further used to optimize the parameters of PID controllers (including the proportional gain, the integral gain, and the differential gain).

The ML approaches are also applied in thermal energy storage systems containing phase-change-materials (PCM) widely used in buildings. For instance, a machine learning exergy-based optimization method is used to optimize the design of a hybrid renewable energy system integrating PCM for active cooling applications (Tang et al., 2020). The machine learning tool is an ANN-based model (an SLFNN model). The input for the SLFNN are geometrical parameters of the system (e.g., pipe diameter, wall thickness, etc.) and optimization parameters (e.g., inlet cooling water temperature, flow rate, etc.). The output from the SLFNN is the system exergy. Then the optimizer based on the teaching-learning-based optimization (TLBO) algorithm (Rao and Patel, 2011) coupled with the trained SLFNN are used to find the optimized design parameters. It is shown that the exergy of the system can be improved by 2.6% (from 849.9 kW to 872.06 kW) when using the optimized design parameters. In (Zhou et al., 2020e), a generic optimization method (integrated with an SLFNN model and heuristic optimization algorithms) is used to optimize the design of a hybrid renewable system integrating PCM for five different climate regions. The input for the SLFNN are eight design geometrical and operational parameters such as the diameter, the mass flow rate, and the inlet cooling water temperature, etc. The output of the SLFNN is the equivalent overall energy generation. The trained SLFNN model is then coupled into the optimizer (based on the TLBO algorithm) to optimize the design parameters. It is shown that the optimization method can help significantly increase the annual equivalent energy output of the system (e.g., increase by 7.4% and 4.2% in two representative cities).

## Summary and future directions

This paper provides a comprehensive review of the application of machine learning technologies in the development and management of energy storage devices and energy storage systems. Machine learning has demonstrated success for solving a range of problems, including state estimation, life prediction, fault and defect diagnosis, property and behavior analysis, as well as modeling, design and optimization. Even more progress is achieved for ESDs with broad commercial applications (including batteries, capacitor/supercapacitors, fuel cells) because of the availability of a large amount of dataset to train the ML models.

Our review shows that the commonly used ML technologies applied in the research and development of ESDs include unsupervised learning, supervised learning (including SVM/SVR, RVM, DT/RF, and regression algorithms), and deep learning (including EML, ANN, CNN, and RNN-based models). Some ML technologies combine multiple algorithms and models (such as combining CNN and RNN, combining RNN and DNN) to achieve the highest learning performance. Among the commonly used machine learning models, the linear regression algorithms have the advantages of simple model structure and high training efficiency based on linear separable datasets; however, they face difficulties in dealing with non-linear datasets and datasets with complex features. Compared with the linear regression algorithms, the SVM/SVR algorithms have advantages in dealing with non-linearly separable datasets, because the data on the original feature space can be projected into a higher space through kernel functions. Besides SVM/SVR, GPR also has the advantage of dealing with non-linearly separable datasets when compared with LR. SVM/SVR usually requires less amount of data to train to achieve a relatively high accuracy compared with deep learning. The drawback of SVM/SVR is that the algorithm is sensitive to kernel selection: an inappropriate kernel function can lead to a failure of training. In addition, SVM/SVR has a low training efficiency when dealing with large datasets. By contrast, the deep learning models have the advantages of high robustness, accuracy, and reliability when dealing with complex problems with multiple features (such as noised datasets, sequenced datasets, and image datasets). These advantages are especially eminent for the deep learning algorithms with complex structures (such as CNN, LSTM, or hybrid deep learning models). The deep learning models also have the advantage of structural flexibility, which can easily suit various types of problem and can overcome the difficulties in kernel selection (such as the drawbacks existed in the GPR, SVM/SVR algorithms). Thus, deep learning has been flourishing in the recent decade and plays an important role in the research and development of ESDs and further ESSs. The drawback of the deep learning algorithm is the requirement of a large amount of training data, especially for CNN- and RNN-based models. This may cause the training efficiency to decrease. On the other hand, a smaller amount of training data may cause a model to fail to capture the required features behind the dataset. Besides, because deep learning models usually have more complicated structures compared with those algorithms with relatively simple and fixed-format structures (such as the regression algorithms), structural designing becomes vital for determining the training efficiency and accuracy of the deep learning models. Often, extra optimization on the model topology is needed. During the training of ML models, the transfer learning and ensemble learning strategies can be applied to increase the training efficiency and model robustness. The common formats of the dataset include discrete data arrays, time-series datasets, and images. Besides, some data-preprocessing technologies, such as data regeneration, normalization, compaction, etc., are very helpful to improve the training accuracy and efficiency. The machine learning approach is a powerful tool in processing and mining multiple formats of dataset to achieve good performance in addressing the problems in the development and management of energy storage devices.

Machine learning technologies are also successfully applied in the development and management of commonly used ESSs, including battery energy storage systems, hybrid energy storage systems (containing at least two modules among batteries, capacitors/supercapacitors, and fuel cells), grids and microgrids containing energy storage modules, and other ESSs (including the pump-storage system and the thermal ESS). For ESS, machine learning mainly focuses on ESS management (such as the energy flow among the ESS units, the energy/power generation/consumption of ESS units, the operational strategies of the energy storage units) and the analysis, design, and optimization (such as the parametric structure design) of the ESS. The supervised learning and deep learning approaches are mostly used to construct regression models for ESS control and ESS design and optimization. The control process is an interaction among the units within the system. Reinforcement learning is widely used in control strategy optimization for the ESS. The commonly used algorithms include *Q*-learning and deep *Q*-networks.

There are still limitations in the application of machine learning for the development and management of ESDs and ESSs. We propose the following four areas for potential future research directions:

1) The machine learning models and algorithms can be further developed and optimized to suit the requirement of the energy storage devices and systems, such as maintaining higher learning accuracy and higher training efficiency when importing a large amount of data containing sophisticated features. Combinations of more types of machine learning models and algorithms can be further developed for data-preprocessing and data mining, such as distilling the features from image clusters with time-series characteristics, which can be beneficial in training the ML model with high accuracy and making optimized decisions. In addition, more advanced ML model construction and training strategies, such as transfer learning, ensemble learning, and pre-training strategies, can be applied to further improve the training accuracy, training efficiency, and model robustness.
2) The machine learning technologies can be applied to more areas beyond the current focus. Although current researches have covered many aspects of the development and management of ESDs and ESSs, there are still many areas which are not well addressed, such as mathematical and physical-based model development, large-scale design and optimization, degradation mechanism analysis, failure detection and prediction, as well as the connection between the micro or nanoscale material characteristics to the macro-scale performance. Many of these are challenging for traditional experimental or numerical approaches, whereas machine learning can be a powerful tool in solving these difficulties. In addition, sufficient relevant data are needed for training the machine learning models (Aykol et al., 2020), in order to promote the training accuracy and reliability of the machine learning model.
3) The machine learning technologies can be coupled with other approaches (such as experiments and numerical simulations) more tightly during the development of energy storage. For instance, machine learning can be used as an intermediate step for processing the experimental or numerical data. This will assist the design of the next-step experiments or simulation strategies, which can reduce redundant experiments or simulations and accelerate the development. A typical example of this aspect is to apply machine learning to reduce the number and duration of the experiments required (Attia et al., 2020), which optimizes the parameter space and promotes the efficiency of development (e.g., battery design, material selection, cell manufacture and control, elongation of the battery lifetime, etc.).
4) Smart energy storage systems based on a high level of artificial intelligence can be developed. With the widespread use of the internet of things (IoT), especially their application in grid management and intelligent vehicles, the demand for the energy use efficiency and fast system response keeps growing. The increasing complexity of the environment makes it more challenging for traditional controlling models to make detections and optimal decisions in a short response period. Besides, the low intelligence of the system increases the working load on the operators, which may cause misoperation to occur frequently and lead to further security risks. As a result, reliable machine learning models and algorithms, which can accurately perceive the environmental features and rapidly make optimized decisions based on the status of each unit is needed in the development and management of energy storage systems. Besides, smart systems containing interactions between ESD and ESS, and use of artificial intelligence to make controls and decisions, are needed. An example of such a smart system based on a high level of artificial intelligence is CHAIN (cyber hierarchy and interactional network) proposed by (Yang et al., 2020a). This system is capable of both interactional computing and data storage and can store abundant information further for deep learning. In addition, improving interpretability and trustworthy of ML in decision-making is needed for ESS management.

## References

[bib2] Andre D., Appel C., Soczka-Guth T., Sauer D.U. (2013). Advanced mathematical methods of SOC and SOH estimation for lithium-ion batteries. J. Power Sources.

[bib3] Attia P.M., Grover A., Jin N., Severson K.A., Markov T.M., Liao Y.-H., Chen M.H., Cheong B., Perkins N., Yang Z. (2020). Closed-loop optimization of fast-charging protocols for batteries with machine learning. Nature.

[bib4] Aykol M., Herring P., Anapolsky A. (2020). Machine learning for continuous innovation in battery technologies. Nat. Rev. Mater..

[bib5] Badmos O., Kopp A., Bernthaler T., Schneider G. (2020). Image-based defect detection in lithium-ion battery electrode using convolutional neural networks. J. Intell. Manuf..

[bib6] Bao J., Murugesan V., Kamp C.J., Shao Y., Yan L., Wang W. (2020). Machine learning coupled multi-scale modeling for redox flow batteries. Adv. Theor. Simul..

[bib7] Biondini G., Vinod Hrishikesh D., Rao C.R. (2015). An introduction to rare event simulation and importance sampling. Handbook of Statistics.

[bib8] Breiman L., Friedman J., Stone C.J., Olshen R.A. (1984). Classification and Regression Trees.

[bib9] Bui V.H., Hussain A., Kim H.M. (2019). Q-learning-based operation strategy for community battery energy storage system (CBESS) in microgrid system. Energies.

[bib10] Cai W., Lesnik K.L., Wade M.J., Heidrich E.S., Wang Y., Liu H. (2019). Incorporating microbial community data with machine learning techniques to predict feed substrates in microbial fuel cells. Biosens. Bioelectron..

[bib11] Carleo G., Cirac I., Cranmer K., Daudet L., Schuld M., Tishby N., Vogt-Maranto L., Zdeborová L. (2019). Machine learning and the physical sciences. Rev. Mod. Phys..

[bib12] Chang Y., Fang H., Zhang Y. (2017). A new hybrid method for the prediction of the remaining useful life of a lithium-ion battery. Appl. Energy.

[bib13] Chaoui H., Gualous H., Boulon L., Kelouwani S. (2019). Deep reinforcement learning energy management system for multiple battery based electric vehicles. 2018 IEEE Veh. Power Propuls. Conf. VPPC 2018.

[bib14] Chawla N.V., Bowyer K.W., Hall L.O., Kegelmeyer W.P. (2002). SMOTE: synthetic minority over-sampling technique. J. Artif. Intell. Res..

[bib15] Chemali E., Kollmeyer P.J., Preindl M., Emadi A. (2018). State-of-charge estimation of Li-ion batteries using deep neural networks: a machine learning approach. J. Power Sources.

[bib16] Chen C., Zuo Y., Ye W., Li X., Deng Z., Ong S.P. (2020). A critical review of machine learning of energy materials. Adv. Energy Mater..

[bib17] Chen Z., Xiong R., Lu J., Li X. (2018). Temperature rise prediction of lithium-ion battery suffering external short circuit for all-climate electric vehicles application. Appl. Energy.

[bib18] Cheng H., Tan P.-N., Gao J., Scripps J., Ng W.K., Kitsuregawa M., Li J., Chang K. (2006). Multistep-ahead time series prediction. Pacific-Asia Conference on Knowledge Discovery and Data Mining: Advances in Knowledge Discovery and Data Mining.

[bib19] Cheng Y., Zerhouni N., Lu C. (2018). A hybrid remaining useful life prognostic method for proton exchange membrane fuel cell. Int. J. Hydrogen Energy.

[bib20] Chia Y.Y., Lee L.H., Shafiabady N., Isa D. (2015). A load predictive energy management system for supercapacitor-battery hybrid energy storage system in solar application using the Support Vector Machine. Appl. Energy.

[bib1] Alaoui, C., 2019. Hybrid vehicle energy management using deep learning. Proc. - 2019 Int. Conf. Intell. Syst. Adv. Comput. Sci. ISACS 2019 1–5. 10.1109/ISACS48493.2019.9068880

[bib21] Cho, K., Van Merriënboer, B., Gulcehre, C., Bahdanau, D., Bougares, F., Schwenk, H., Bengio, Y., 2014. Learning phrase representations using RNN encoder-decoder for statistical machine translation. EMNLP 2014 - 2014 Conf. Empir. Methods Nat. Lang. Process. Proc. Conf. 1724–1734. 10.3115/v1/d14-1179

[bib22] Chollet, F., 2017. Xception: Deep learning with depthwise separable convolutions, in: Proceedings of the IEEE Conference on Computer Vision and Pattern Recognition. pp. 1251–1258.

[bib24] Deng C., Ji X., Rainey C., Zhang J., Lu W. (2020). Integrating machine learning with human knowledge. iScience.

[bib25] Dong L., Wesseloo J., Potvin Y., Li X. (2016). Discrimination of mine seismic events and blasts using the Fisher classifier , naive Bayesian classifier and logistic regression. Rock Mech. Rock Eng..

[bib26] Dongale T.D., Jadhav P.R., Navathe G.J., Kim J.H., Karanjkar M.M., Patil P.S. (2015). Development of nano fiber MnO_2_ thin film electrode and cyclic voltammetry behavior modeling using artificial neural network for supercapacitor application. Mater. Sci. Semicond. Process..

[bib27] Dozat T. (2016). Incorporating nesterov momentum into adam. Proceedings of 4th International Conference on Learning Representations.

[bib28] Dunjko V., Briegel H.J. (2018). Machine learning & artificial intelligence in the quantum domain: a review of recent progress. Rep. Prog. Phys..

[bib29] Erick, A.O. and Folly, K.A., 2020. Reinforcement learning approaches to power management in grid-tied microgrids: A review, in: 2020 Clemson University Power Systems Conference (PSC). IEEE, pp. 1–6.

[bib30] Ester M., Kriegel H.-P., Sander J., Xu X. (1996). A density-based algorithm for discovering clusters in large spatial databases with noise.

[bib31] Estrach J.B., Szlam A., LeCun Y. (2014). Signal recovery from pooling representations. International Conference on Machine Learning.

[bib32] Feng C. (2019). Controller optimization approach using LSTM-based identification model for pumped-storage units. IEEE Access.

[bib33] Fischer A., Igel C. (2012). An introduction to restricted Boltzmann machines. Iberoamerican Congress on Pattern Recognition.

[bib34] Flah M., Nunez I., Ben W., Moncef C. (2020). Machine learning algorithms in civil structural health monitoring: a systematic review. Arch. Comput. Methods Eng..

[bib35] Foiadelli F., Longo M., Miraftabzadeh S. (2018). Energy consumption prediction of electric vehicles based on big data approach. Proc. - 2018 IEEE Int. Conf. Environ. Electr. Eng. 2018 IEEE Ind. Commer. Power Syst. Eur. EEEIC/I CPS Eur. 2018.

[bib36] Gao T., Lu W. (2020). Physical model and machine learning enabled electrolyte channel design for fast charging. J. Electrochem. Soc..

[bib37] Gao T., Ying L., Dai M., Shen G., Hu P., Shen L. (2019). A comparative study of temperature-dependent interfacial heat transfer coefficient prediction methods for 22MnB5 steel in spray quenching process. Int. J. Therm. Sci..

[bib38] Gayon-Lombardo A., Mosser L., Brandon N.P., Cooper S.J. (2020). Pores for thought: generative adversarial networks for stochastic reconstruction of 3D multi-phase electrode microstructures with periodic boundaries. NPJ Comput. Mater..

[bib39] Ghasemi M., Nassef A.M., Al-Dhaifallah M., Rezk H. (2020). Performance improvement of microbial fuel cell through artificial intelligence. Int. J. Energy Res..

[bib40] Goh G.B., Hodas N.O., Vishnu A. (2017). Deep learning for computational chemistry. J. Comput. Chem..

[bib41] Goodfellow I., Bengio Y., Courville A., Bengio Y. (2016).

[bib42] Goodfellow I., Pouget-Abadie J., Mirza M., Xu B., Warde-Farley D., Ozair S., Courville A., Bengio Y., Ghahramani Z., Welling M., Cortes C., Lawrence N.D., Weinberger K.Q. (2014). Generative adversarial nets. Advances in Neural Information Processing Systems.

[bib43] Gou B., Xu Y., Fang S., Pratama R.A., Liu S. (2019). Remaining useful life prediction for lithium-ion battery using ensemble learning method. IEEE Power Energy Soc. Gen. Meet. 2019-Augus.

[bib44] Gu G.H., Noh J., Kim I., Jung Y. (2019). Machine learning for renewable energy materials. J. Mater. Chem. A.

[bib45] Haider S.N., Zhao Q., Li X. (2020). Data driven battery anomaly detection based on shape based clustering for the data centers class. J. Energy Storage.

[bib46] He Y., Ma Z. (2016). A data-driven Gaussian process regression model for two-chamber microbial fuel cells. Fuel Cells.

[bib47] Hinton G.E., Osindero S., Teh Y.-W. (2006). A fast learning algorithm for deep belief nets. Neural Comput..

[bib48] Hochreiter S. (1997). Long short-term memory. Neural Comput..

[bib49] Hu X., Xu L., Lin X., Pecht M. (2020). Battery lifetime prognostics. Joule.

[bib50] Hua H., Qin Y., Hao C., Cao J. (2019). Optimal energy management strategies for energy Internet via deep reinforcement learning approach. Appl. Energy.

[bib51] Huang G.-B., Zhou H., Ding X., Zhang R. (2011). Extreme learning machine for regression and multiclass classification. IEEE Trans. Syst. Man. Cybern. B Cybern..

[bib52] Huang X., Ye Y., Xiong L., Lau R.Y.K., Jiang N., Wang S. (2016). Time series k -means: a new k -means type smooth subspace clustering for time series data. Inf. Sci. (NY).

[bib53] Jain R.K., Smith K.M., Culligan P.J., Taylor J.E. (2014). Forecasting energy consumption of multi-family residential buildings using support vector regression: Investigating the impact of temporal and spatial monitoring granularity on performance accuracy. Appl. Energy.

[bib54] Julier S.J., Uhlmann J.K., Kadar K. (1997). New extension of the Kalman filter to nonlinear systems. Signal Processing, Sensor Fusion, and Target Recognition VI.

[bib55] Kalman R.E. (1960). A new approach to linear filtering and prediction problems. J. Basic Eng..

[bib56] Khumprom P., Yodo N. (2019). A data-driven predictive prognostic model for lithium-ion batteries based on a deep learning algorithm. Energies.

[bib57] Kim D.H., Kim T.J.Y., Wang X., Kim M., Quan Y.J., Oh J.W., Min S.H., Kim H., Bhandari B., Yang I., Ahn S.H. (2018). Smart machining process using machine learning: a review and perspective on machining industry. Int. J. Precis. Eng. Manuf. Green Technol..

[bib58] Kim S., Lee S., Cho K. (2012). Design of high-performance unified circuit for linear and non-linear SVM classifications. J. Semicond. Tech. Sci..

[bib59] Kingma D.P., Ba J. (2014). Adam: a method for stochastic optimization. arxiv.org/abs/1412.6980.

[bib60] Kofinas P., Dounis A.I., Vouros G.A. (2018). Fuzzy Q-Learning for multi-agent decentralized energy management in microgrids. Appl. Energy.

[bib61] Kofinas P., Vouros G., Dounis A.I. (2018). Energy management in solar microgrid via reinforcement learning using fuzzy reward. Adv. Build. Energy Res..

[bib62] Lee S., Choi D.H. (2020). Energy management of smart home with home appliances, energy storage system and electric vehicle: a hierarchical deep reinforcement learning approach. Sensors (Switzerland).

[bib63] Lee S., Choi D.H. (2019). Reinforcement learning-based energy management of smart home with rooftop solar photovoltaic system, energy storage system, and home appliances. Sensors (Switzerland).

[bib64] Lesnik K.L., Liu H. (2017). Predicting microbial fuel cell biofilm communities and bioreactor performance using artificial neural networks. Environ. Sci. Technol..

[bib65] Li S., He H., Li J. (2019). Big data driven lithium-ion battery modeling method based on SDAE-ELM algorithm and data pre-processing technology. Appl. Energy.

[bib66] Li, S., Li, J., Wang, H., 2019b. Big data driven Lithium-ion battery modeling method: a cyber-physical system approach. Proc. - 2019 IEEE Int. Conf. Ind. Cyber Phys. Syst. ICPS 2019 161–166. 10.1109/ICPHYS.2019.8780152

[bib67] Li X., Zhang T., Liu Y. (2019). Detection of voltage anomalies in spacecraft storage batteries based on a deep belief network. Sensors (Switzerland).

[bib68] Li Y., Zou C., Berecibar M., Nanini-Maury E., Chan J.C.W., van den Bossche P., Van Mierlo J., Omar N. (2018). Random forest regression for online capacity estimation of lithium-ion batteries. Appl. Energy.

[bib70] Liu C., Tan J., Shi H., Wang X. (2018). Lithium-ion cell screening with convolutional neural networks based on two-step time-series clustering and hybrid resampling for imbalanced data. IEEE Access.

[bib71] Liu D., Zhou J., Pan D., Peng Y., Peng X. (2015). Lithium-ion battery remaining useful life estimation with an optimized Relevance Vector Machine algorithm with incremental learning. Meas. J. Int. Meas. Confed..

[bib72] Liu J., Chen Z. (2019). Remaining useful life prediction of lithium-ion batteries based on health indicator and Gaussian process regression model. IEEE Access.

[bib73] Liu J., Li Q., Chen W., Yan Y., Qiu Y., Cao T. (2019). Remaining useful life prediction of PEMFC based on long short-term memory recurrent neural networks. Int. J. Hydrogen Energy.

[bib74] Liu Y., Zhao G., Peng X. (2019). Deep learning prognostics for lithium-ion battery based on ensembled long short-term memory networks. IEEE Access.

[bib75] Lokhande P.E. (2020). Cyclic voltammetry behavior modeling of fabricated nanostructured Ni(OH)_2_ electrode using artificial neural network for supercapacitor application. Proc. Inst. Mech. Eng. C.

[bib76] Lucu M., Martinez-Laserna E., Gandiaga I., Liu K., Camblong H., Widanage W.D., Marco J. (2020). Data-driven nonparametric Li-ion battery ageing model aiming at learning from real operation data - Part B: cycling operation. J. Energy Storage.

[bib77] Ma G., Zhang Y., Cheng C., Zhou B., Hu P., Yuan Y. (2019). Remaining useful life prediction of lithium-ion batteries based on false nearest neighbors and a hybrid neural network. Appl. Energy.

[bib78] Ma J., Liu X., Zou X., Yue M., Shang P., Kang L., Jemei S., Lu C., Ding Y., Zerhouni N., Cheng Y. (2020). Degradation prognosis for proton exchange membrane fuel cell based on hybrid transfer learning and intercell differences. ISA Trans..

[bib79] Ma L., Xie W., Zhan g.Y. (2019). Blister defect detection based on convolutional neural network for polymer lithium-ion battery. Appl. Sci..

[bib80] Mbuwir B.V., Kaffash M., Deconinck G. (2018). Battery scheduling in a residential multi-carrier energy system using reinforcement learning. 2018 IEEE Int. Conf. Commun. Control. Comput. Technol. Smart Grids, SmartGridComm 2018.

[bib81] McCoy J.T., Auret L. (2019). Machine learning applications in minerals processing: a review. Miner. Eng..

[bib82] Mehnatkesh H., Alasty A., Boroushaki M., Khodsiani M.H., Hasheminasab M.R., Kermani M.J. (2020). Estimation of water coverage ratio in low temperature PEM-fuel cell using deep neural network. IEEE Sens. J..

[bib83] Mejia C., Kajikawa Y. (2020). Emerging topics in energy storage based on a large-scale analysis of academic articles and patents. Appl. Energy.

[bib84] Meng H., Li Y.F. (2019). A review on prognostics and health management (PHM) methods of lithium-ion batteries. Renew. Sustain. Energy Rev..

[bib85] Morando S., Jemei S., Hissel D., Gouriveau R., Zerhouni N. (2017). Proton exchange membrane fuel cell ageing forecasting algorithm based on Echo State Network. Int. J. Hydrogen Energy.

[bib86] Moré J.J., Watson G.A. (1978). The Levenberg-Marquardt algorithm: implementation and theory. Numerical Analysis.

[bib87] Morgan D., Jacobs R. (2020). Opportunities and challenges for machine learning in materials science. Annu. Rev. Mater. Res..

[bib88] Murnane, M., Ghazel, A., 2017. A closer look at state of charge (SOC) and state of health (SOH) estimation techniques for batteries. Analog devices. https://www.analog.com/media/en/technical-documentation/technical-articles/a-closer-look-at-state-of-charge-and-state-health-estimation-techniques.pdf (accessed July 3, 2017).

[bib89] Ng M.-F., Zhao J., Yan Q., Conduit G.J., Seh Z.W. (2020). Predicting the state of charge and health of batteries using data-driven machine learning. Nat. Mach. Intell..

[bib90] Ortiz J.P., Valladolid J.D., Garcia C.L., Novillo G., Berrezueta F. (2019). Analysis of machine learning techniques for the intelligent diagnosis of Ni-MH battery cells. 2018 IEEE Int. Autumn Meet. Power, Electron. Comput. ROPEC 2018.

[bib91] Pan H., Lü Z., Wang H., Wei H., Chen L. (2018). Novel battery state-of-health online estimation method using multiple health indicators and an extreme learning machine. Energy.

[bib92] Panteleev J., Gao H., Jia L. (2018). Recent applications of machine learning in medicinal chemistry. Bioorg. Med. Chem. Lett..

[bib93] Park K., Choi Y., Choi W.J., Ryu H.Y., Kim H. (2020). LSTM-based battery remaining useful life prediction with multi-channel charging profiles. IEEE Access.

[bib94] Parwaiz S., Malik O.A., Pradhan D., Khan M.M. (2018). Machine-learning-based cyclic voltammetry behavior model for supercapacitance of Co-doped ceria/rGO nanocomposite. J. Chem. Inf. Model.

[bib95] Patel A.G., Johnson L., Arroyave R., Lutkenhaus J.L. (2019). Design of multifunctional supercapacitor electrodes using an informatics approach. Mol. Syst. Des. Eng..

[bib96] Patil M.A., Tagade P., Hariharan K.S., Kolake S.M., Song T., Yeo T., Doo S. (2015). A novel multistage Support Vector Machine based approach for Li ion battery remaining useful life estimation. Appl. Energy.

[bib166] Pollet B.G., Staffell I., Shang J.L., Molkov V., Folkson R. (2014). Fuel-cell (hydrogen) electric hybrid vehicles. Alternative Fuels and Advanced Vehicle Technologies for Improved Environmental Performance.

[bib98] Pozo B., Garate J.I., Ferreiro S., Fernandez I., Fernandez de Gorostiza E. (2018). Supercapacitor electro-mathematical and machine learning modelling for low power applications. Electron.

[bib99] Qu J., Liu F., Ma Y., Fan J. (2019). A neural-network-based method for RUL prediction and SOH monitoring of lithium-ion battery. IEEE Access.

[bib100] Rao R.V., Patel V. (2011). Thermodynamic optimization of plate-fin heat exchanger using teaching-learning-based optimization (TLBO) algorithm. Int. J. Adv. Manuf. Technol..

[bib101] Reddy N.P., Pasdeloup D., Zadeh M.K., Skjetne R. (2019). An intelligent power and energy management system for fuel cell/battery hybrid electric vehicle using reinforcement learning. ITEC 2019 - 2019 IEEE Transp. Electrif. Conf. Expo..

[bib102] Ren J., Lin X., Liu J., Han T., Wang Z., Zhang H., Li J. (2020). Engineering early prediction of supercapacitors’ cycle life using neural networks. Mater. Today Energy.

[bib103] Ren L., Zhao L., Hong S., Zhao S., Wang H., Zhang L. (2018). Remaining useful life prediction for lithium-ion battery: a deep learning approach. IEEE Access.

[bib104] Richardson R.R., Birkl C.R., Osborne M.A., Howey D.A. (2018). Gaussian process regression for in situ capacity estimation of lithium-ion batteries. IEEE Trans. Ind. Inform..

[bib105] Richardson R.R., Osborne M.A., Howey D.A. (2017). Gaussian process regression for forecasting battery state of health. J. Power Sources.

[bib106] Sadowsky J.S. (1990). A new method for Viterbi decoder simulation using importance sampling. IEEE Trans. Commun..

[bib107] Sahinoglu G.O., Pajovic M., Sahinoglu Z., Wang Y., Orlik P.V., Wada T. (2017). Battery state-of-charge estimation based on regular/recurrent Gaussian process regression. IEEE Trans. Ind. Electron..

[bib108] Severson K.A., Attia P.M., Jin N., Perkins N., Jiang B., Yang Z., Chen M.H., Aykol M., Herring P.K., Fraggedakis D. (2019). Data-driven prediction of battery cycle life before capacity degradation. Nat. Energy.

[bib109] Shen S., Sadoughi M., Li M., Wang Z., Hu C. (2020). Deep convolutional neural networks with ensemble learning and transfer learning for capacity estimation of lithium-ion batteries. Appl. Energy.

[bib110] Silva J.C.F., Teixeira R.M., Silva F.F., Brommonschenkel S.H., Fontes E.P.B. (2019). Machine learning approaches and their current application in plant molecular biology: a systematic review. Plant Sci..

[bib111] Simonyan K., Zisserman A. (2014). Very deep convolutional networks for large-scale image recognition. arxiv.org/abs/1409.1556.

[bib112] Song X., Yang F., Wang D., Tsui K.L. (2019). Combined CNN-LSTM network for state-of-charge estimation of lithium-ion batteries. IEEE Access.

[bib113] Srivastava N., Hinton G., Krizhevsky A., Sutskever I., Salakhutdinov R. (2014). Dropout: a simple way to prevent neural networks from overfitting. J. Mach. Learn. Res..

[bib114] Su H., Lian C., Liu J., Liu H. (2019). Machine learning models for solvent effects on electric double layer capacitance. Chem. Eng. Sci..

[bib115] Sun H., Fu Z., Tao F., Zhu L., Si P. (2020). Data-driven reinforcement-learning-based hierarchical energy management strategy for fuel cell/battery/ultracapacitor hybrid electric vehicles. J. Power Sources.

[bib116] Szegedy, C., Vanhoucke, V., Ioffe, S., Shlens, J., Wojna, Z., 2016. Rethinking the inception architecture for computer vision, in: Proceedings of the IEEE Conference on Computer Vision and Pattern Recognition. pp. 2818–2826.

[bib117] Takagishi Y., Yamanaka T., Yamaue T. (2019). Machine learning approaches for designing mesoscale structure of li-ion battery electrodes. Batteries.

[bib118] Tan Y., Matsui H., Ishiguro N., Uruga T., Nguyen D.N., Sekizawa O., Sakata T., Maejima N., Higashi K., Dam H.C., Tada M. (2019). Pt-Co/C cathode catalyst degradation in a polymer electrolyte fuel cell investigated by an infographic approach combining three-dimensional spectroimaging and unsupervised learning. J. Phys. Chem. C.

[bib119] Tang J., Huang P. (2019). Modeling the electrical conductivity of anode for solid oxide fuel cell using support vector regression machine. IOP Conf. Ser. Mater. Sci. Eng..

[bib120] Tang L., Zhou Y., Zheng S., Zhang G. (2020). Exergy-based optimisation of a phase change materials integrated hybrid renewable system for active cooling applications using supervised machine learning method. Sol. Energy.

[bib121] Tang X., Yao K., Liu B., Hu W., Gao F. (2018). Long-term battery voltage, power, and surface temperature prediction using a model-based extreme learning machine. Energies.

[bib122] Tipping M.E., Bousquet O., von Luxburg U., Ratsch G. (2003). Bayesian inference: an introduction to principles and practice in machine learning. Advanced Lectures on Machine Learning.

[bib123] Tipping M.E. (2001). Sparse Bayesian learning and the relevance vector machine. J. Mach. Learn. Res..

[bib124] Tong S., Lacap J.H., Park J.W. (2016). Battery state of charge estimation using a load-classifying neural network. J. Energy Storage.

[bib125] Tsompanas M.A., You J., Wallis L., Greenman J., Ieropoulos I. (2019). Artificial neural network simulating microbial fuel cells with different membrane materials and electrode configurations. J. Power Sources.

[bib126] Veeraraghavan, A., Adithya, V., Bhave, A., Akella, S., 2018. Battery aging estimation with deep learning. 2017 IEEE Transp. Electrif. Conf. ITEC-India 2017 2018-Janua, 1–4. 10.1109/ITEC-India.2017.8333827

[bib127] Wang B., Xie B., Xuan J., Jiao K. (2020). AI-based optimization of PEM fuel cell catalyst layers for maximum power density via data-driven surrogate modeling. Energy Convers. Manag..

[bib129] Wang, Y., Chen, Y., Liao, X., Dong, L., 2019. Lithium-ion Battery Face Imaging with Contactless Walabot and Machine Learning. Proc. 2019 IEEE Int. Conf. Mechatronics Autom. ICMA 2019 1067–1072. 10.1109/ICMA.2019.8816512

[bib128] Wang J., Liu P., Hicks-Garner J., Sherman E., Soukiazian S., Verbrugge M., Tataria H., Musser J., Finamore P. (2011). Cycle-life model for graphite-LiFePO_4_ cells. J. Power Sources.

[bib130] Wang Z., Qu J., Fang X., Li H., Zhong T., Ren H. (2020). Prediction of early stabilization time of electrolytic capacitor based on ARIMA-Bi_LSTM hybrid model. Neurocomputing.

[bib131] Wang Z., Wang P. (2014). A maximum confidence enhancement based sequential sampling scheme for simulation-based design. J. Mech. Des..

[bib133] Wu J., Zhang C., Chen Z. (2016). An online method for lithium-ion battery remaining useful life estimation using importance sampling and neural networks. Appl. Energy.

[bib134] Wu N., Wang H. (2018). Deep learning adaptive dynamic programming for real time energy management and control strategy of micro-grid. J. Clean. Prod..

[bib132] Wu B., Han S., Shin K.G., Lu W. (2018). Application of artificial neural networks in design of lithium-ion batteries. J. Power Sources.

[bib135] Wu Y., Tan H., Peng J., Zhang H., He H. (2019). Deep reinforcement learning of energy management with continuous control strategy and traffic information for a series-parallel plug-in hybrid electric bus. Appl. Energy.

[bib136] Xiao B., Liu Y., Xiao B. (2019). Accurate state-of-charge estimation approach for lithium-ion batteries by gated recurrent unit with ensemble optimizer. IEEE Access.

[bib137] Xiong R., Cao J., Yu Q. (2018). Reinforcement learning-based real-time power management for hybrid energy storage system in the plug-in hybrid electric vehicle. Appl. Energy.

[bib138] Yang S., He R., Zhang Z., Cao Y., Gao X., Liu X. (2020). CHAIN: cyber hierarchy and interactional network enabling digital solution for battery full-Lifespan management. Matter.

[bib139] Yang Z., Zhu F., Lin F. (2020). Deep-reinforcement-learning-based energy management strategy for supercapacitor energy storage systems in urban rail transit. IEEE Trans. Intell. Transp. Syst..

[bib140] Yao L., Xiao Y., Gong X., Hou J., Chen X. (2020). A novel intelligent method for fault diagnosis of electric vehicle battery system based on wavelet neural network. J. Power Sources.

[bib141] Ying L., Gao T., Dai M., Hu P. (2017). Investigation of interfacial heat transfer mechanism for 7075-T6 aluminum alloy in HFQ hot forming process. Appl. Therm. Eng..

[bib142] Ying L., Gao T., Dai M., Yang Y., Hu P. (2017). Experimental investigation of temperature-dependent interfacial heat transfer mechanism with spray quenching for 22MnB5 steel. Appl. Therm. Eng..

[bib143] You G.W., Park S., Oh D. (2017). Diagnosis of electric vehicle batteries using recurrent neural networks. IEEE Trans. Ind. Electron..

[bib145] Zhang H., Tang W., Na W., Lee P.Y., Kim J. (2020). Implementation of generative adversarial network-CLS combined with bidirectional long short-term memory for lithium-ion battery state prediction. J. Energy Storage.

[bib146] Zhang L., Lin J., Liu B., Zhang Z., Yan X., Wei M. (2019). A review on deep learning applications in prognostics and health management. IEEE Access.

[bib147] Zhang Y., Xiong R., He H., Pecht M.G. (2018). Long short-term memory recurrent neural network for remaining useful life prediction of lithium-ion batteries. IEEE Trans. Veh. Technol..

[bib148] Zhang Z., Li S., Xiao Y., Yang Y. (2019). Intelligent simultaneous fault diagnosis for solid oxide fuel cell system based on deep learning. Appl. Energy.

[bib149] Zhao Y., Liu P., Wang Z., Zhang L., Hong J. (2017). Fault and defect diagnosis of battery for electric vehicles based on big data analysis methods. Appl. Energy.

[bib150] Zheng Z., Chen B., Xu Y., Fritz N., Gurumukhi Y., Cook J., Ates M.N., Miljkovic N., Braun P.V., Wang P. (2020). A Gaussian process-based crack pattern modeling approach for battery anode materials design. J. Electrochem. En. Conv. Stor..

[bib151] Zheng Z., Morando S., Pera M.C., Hissel D., Larger L., Martinenghi R., Baylon Fuentes A. (2017). Brain-inspired computational paradigm dedicated to fault diagnosis of PEM fuel cell stack. Int. J. Hydrogen Energy.

[bib152] Zhou M., Gallegos A., Liu K., Dai S., Wu J. (2020). Insights from machine learning of carbon electrodes for electric double layer capacitors. Carbon N Y.

[bib153] Zhou P., He Z., Han T., Li X., Lai X., Yan L., Lv T., Xie J., Zheng Y. (2020). A rapid classification method of the retired LiCoxNiyMn1−x−yO2 batteries for electric vehicles. Energy Rep..

[bib154] Zhou X., Hsieh S.J., Peng B., Hsieh D. (2017). Cycle life estimation of lithium-ion polymer batteries using artificial neural network and support vector machine with time-resolved thermography. Microelectron. Reliab..

[bib155] Zhou Y., Huang M., Chen Y., Tao Y. (2016). A novel health indicator for on-line lithium-ion batteries remaining useful life prediction. J. Power Sources.

[bib156] Zhou Y., Huang M., Pecht M. (2020). Remaining useful life estimation of lithium-ion cells based on k-nearest neighbor regression with differential evolution optimization. J. Clean. Prod..

[bib157] Zhou Y., Huang Y., Pang J., Wang K. (2019). Remaining useful life prediction for supercapacitor based on long short-term memory neural network. J. Power Sources.

[bib159] Zhou Y., Wang Y., Wang K., Kang L., Peng F., Wang L., Pang J. (2020). Hybrid genetic algorithm method for efficient and robust evaluation of remaining useful life of supercapacitors. Appl. Energy.

[bib160] Zhou Y., Zheng S., Zhang G. (2020). Machine learning-based optimal design of a phase change material integrated renewable system with on-site PV, radiative cooling and hybrid ventilations—study of modelling and application in five climatic regions. Energy.

[bib161] Zhu S., Li J., Ma L., He C., Liu E., He F., Shi C., Zhao N. (2018). Artificial neural network enabled capacitance prediction for carbon-based supercapacitors. Mater. Lett..

[bib162] Zhu S., Sun X., Gao X., Wang J., Zhao N., Sha J. (2019). Equivalent circuit model recognition of electrochemical impedance spectroscopy via machine learning. J. Electroanal. Chem..

[bib163] Zhu S., Zhao N., Sha J. (2019). Predicting battery life with early cyclic data by machine learning. Energy Storage.

[bib164] Zhu Z., Zhu J., Guo X., Jiang Y., Sun Y. (2019). Numerical modeling of suspension force for bearingless flywheel machine based on differential evolution extreme learning machine. Energies.

[bib165] Zitnik M., Nguyen F., Wang B., Leskovec J., Goldenberg A., Hoffman M.M. (2019). Machine learning for integrating data in biology and medicine: principles, practice, and opportunities. Inf. Fusion.

